# Synthetic and Natural Insecticides: Gas, Liquid, Gel and Solid Formulations for Stored-Product and Food-Industry Pest Control

**DOI:** 10.3390/insects12070590

**Published:** 2021-06-29

**Authors:** Vaclav Stejskal, Tomas Vendl, Radek Aulicky, Christos Athanassiou

**Affiliations:** 1 Crop Research Institute, Drnovska 507/73, 16106 Prague, Czech Republic; vendl@vurv.cz (T.V.); aulicky@vurv.cz (R.A.); 2 Laboratory of Entomology and Agricultural Zoology, Department of Agriculture, Crop Production and Rural Environment, University of Thessaly, Phytokou Str., 38446 Nea Ionia, Greece; athanassiou@agr.uth.gr

**Keywords:** IPM, insecticides, spray, aerosol, baits, fumigation, impregnated nets, essential oils, diatomaceous earth, nanoparticles

## Abstract

**Simple Summary:**

Currently, there is great global research interest in the use of novel methods of protection against agricultural, storage, and urban pests, particularly in the use of botanical substances and nontoxic materials. To ensure efficacy and safety, botanical and synthetic insecticides must be properly formulated and delivered in a species-specific way to their pest targets. The wide diversity of pests and environments—globally occurring at farms and food industry facilities—has inevitably resulted in a massive proliferation of application formulations and approaches. Although there are excellent summaries on particular aspects of the usage of synthetic and botanical pesticides, a general overview of application formulations on stored-product and food-associated pests is not currently available. This review provides an inventory of current and historical pesticide formulations. Its structure follows the traditional insecticide categorization based on four physical formulation types: gas, liquid, gel/foam, and solid. The review documents renewed research interest in optimizing traditional methods, such as insecticide baits, aerosols, sprays, fumigants, and inert gases, as well as the feasibility of integrating these methods with natural insecticides and physical measures (e.g., low temperatures) as combined application approaches. Several emerging technologies of pesticide formulations have been identified; they include electrostatic dusts or sprays, nanoparticles, hydrogels, inert baits with synthetic attractants, biodegradable cyanogenic protective coatings of grain, and RNA-based gene silencing compounds encapsulated in baits. Traditional and new formulations of natural compounds, including inert dust (diatomaceous earth) and botanicals (essential oils), have been considered as non-synthetic chemical control solutions for organic food production in developed countries and as affordable home-made insecticides in developing countries. The authors hope that the general coverage and extensive photographic documentation will make this review useful not only for scientists but also for students and practitioners.

**Abstract:**

The selective application of insecticides is one of the cornerstones of integrated pest management (IPM) and management strategies for pest resistance to insecticides. The present work provides a comprehensive overview of the traditional and new methods for the application of gas, liquid, gel, and solid physical insecticide formulations to control stored-product and food industry urban pests from the taxa Acarina, Blattodea, Coleoptera, Diptera, Hymenoptera, Lepidoptera, Psocoptera, and Zygentoma. Various definitions and concepts historically and currently used for various pesticide application formulations and methods are also described. This review demonstrates that new technological advances have sparked renewed research interest in the optimization of conventional methods such as insecticide aerosols, sprays, fumigants, and inert gases. Insect growth regulators/disruptors (IGRs/IGDs) are increasingly employed in baits, aerosols, residual treatments, and as spray-residual protectants for long-term stored-grain protection. Insecticide-impregnated hypoxic multilayer bags have been proven to be one of the most promising low-cost and safe methods for hermetic grain storage in developing countries. Insecticide-impregnated netting and food baits were originally developed for the control of urban/medical pests and have been recognized as an innovative technology for the protection of stored commodities. New biodegradable acaricide gel coatings and nets have been suggested for the protection of ham meat. Tablets and satchels represent a new approach for the application of botanicals. Many emerging technologies can be found in the form of impregnated protective packaging (insect growth regulators/disruptors (IGRs/IGDs), natural repellents), pheromone-based attracticides, electrostatic dust or sprays, nanoparticles, edible artificial sweeteners, hydrogels, inert baits with synthetic attractants, biodegradable encapsulations of active ingredients, and cyanogenic protective grain coatings. Smart pest control technologies based on RNA-based gene silencing compounds incorporated into food baits stand at the forefront of current strategic research. Inert gases and dust (diatomaceous earth) are positive examples of alternatives to synthetic pesticide products, for which methods of application and their integration with other methods have been proposed and implemented in practice. Although many promising laboratory studies have been conducted on the biological activity of natural botanical insecticides, published studies demonstrating their effective industrial field usage in grain stores and food production facilities are scarce. This review shows that the current problems associated with the application of some natural botanical insecticides (e.g., sorption, stability, field efficacy, and smell) to some extent echo problems that were frequently encountered and addressed almost 100 years ago during the transition from ancient to modern classical chemical pest control methods.

## 1. Formulations Are Adapted According to Specific Routes of Insect Body Entry, Arthropod Diversity, and Various Environmental Conditions

At the worldwide scale, stored-product, urban, and food industry pests annually cause substantial damage to stored commodities and processed food due to direct feeding losses [1,2,3] or contamination by allergens [4,5]. These negative impacts should be systematically diminished through the implementation of integrated pest management (IPM) programs and the establishment of an effective quarantine network helping to reduce pest spread via infested freight containers [6] or other transport means used during national and international trading of commodities [7]. In addition to physical and biological methods, the selective, targeted, and effective use of synthetic or natural insecticides is still the cornerstone of most IPM programs and strategies for insecticide resistance management (IRM) [8,9,10,11,12].

**Figure 1:**
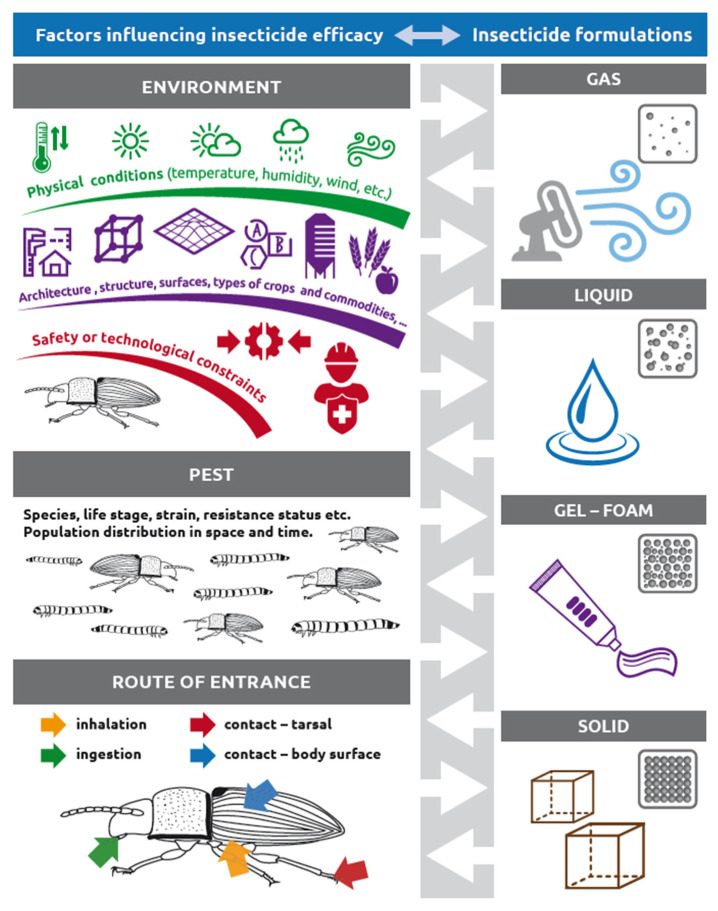
The selection and efficacy of a particular insecticide formulation is affected by a complex interplay between multiple factors that include insecticides, environment (temperature, humidity, crop, structure of stores, technology), workplace and environmental safety and technological constraints, and pests (species, stage, resistance, targeted route of entry into the insect body, etc.).

The biological effect of an insecticide primarily depends on the activity of the active substance (toxicity, hormonal or behavioral disruption, etc.) against the target arthropods. However, to ensure good field efficacy of insecticides, the active substance must be properly formulated both physically and chemically [13,14] and then delivered in sufficient quantity and in the most active form to the physiologically sensitive target site of the arthropod [15]. Depending on the conditions, various physical formulations of identical chemically active ingredients may have different biological activities on identical pest species or their various populations (resistant/sensitive). Physical formulations are adapted according to specific routes of insect body entry, pest-arthropod species biology/ecology, and various environmental conditions (Figure 1).

Formulations of insecticides are traditionally [16,17] divided according to the specific routes of entry [18,19] and their physiological effects, such as stomach poisons, contact poisons, and fumigants. The main routes of insecticide entry to the arthropod body are visualized in Figure 1 and include the oral-digestive route (digestive tract), the dermal-contact route (tarsi, antennae, or the entire surface of the cuticle and intersegmental membranes), and the respiratory-inhalation route (spiracle and insect tracheal system, mite integument). The main routes of entry and their physiological effects are traditionally associated with certain physicochemical formulations of insecticides, such as gaseous (fumigants and vapors), liquid (sprays/aerosols), solid (dusts), and gel or foam (baits) insecticide forms [20,21]. Currently, there exists a conspicuously high degree of diversity of insecticide physical formulations and variance in their usage at both global and local scales. Presumably, this phenomenon can be explained by the technological adaptation of pesticide formulations to various pest body entry routes and the complex environmental and social conditions in which they are applied [22,23]. For example, various insecticide formulations may require different temperatures above certain minimum thresholds that are associated with sufficient respiratory activity (e.g., fumigants or modified atmospheres) or locomotor activity (e.g., baits) of the target pest-arthropod species [24,25]. The enormous diversity of the species of storage- and food-associated pests [22] and variance in their biological (e.g., resistance), ecological, and ethological properties inevitably led to the gradual development of a rich spectrum of more or less species-specific insecticide formulations and methods of their application [26,27,28]. Pesticide product proliferation also reflects the geographical variability and specificity of local social and economic conditions [24,29,30]. Sparks et al. [31] mentioned that the diversity of approaches employed in the insecticide discovery process (e.g., competitor-inspired products, bioactive hypotheses, and natural products) has also profoundly contributed to the discovery of new classes and formulations of insecticides.

Several published reviews, books, and manuals are available either on spraying equipment and application machinery or on insecticide chemical formulations and their active ingredients [27,32,33,34,35,36,37,38,39,40]. However, a comprehensive overview of insecticide formulations based on their physical state during application is missing. Therefore, the present work aimed to develop an inventory of traditional and new methods for the application of various insecticide formulations to control stored-product and food industry (urban) pests, such as mites (Acari), beetles (Coleoptera), moths (Lepidoptera), flies (Diptera), psocids (Psocoptera), ants and wasps (Hymenoptera), cockroaches (Blattodea), crickets (Orthoptera), and silverfishes (Zygentoma) [1,41,42]. In this review, the main chapters follow the traditional insecticide categorization based on four physical formulation types (gas and vapour, liquid, gel and foam, and solid), as summarized by the infographic in Figure 1 and Table 1. Cold plasma, as the remaining physical state of matter, has also been tested for the control of stored-product pests [43]. However, plasma is not included in this review since, to our knowledge, it is not currently classified as a chemical pesticide. We mostly review the application of formulations and treatment methods for the control of stored-product and urban pest arthropods as related to grain stores, food production facilities, and food distribution/retail chains [44]. However, additional knowledge and inspiration are included in this review from general or specific literature on pest control technologies and application methods. Since there is renewed interest in the older pest control technologies that we included, where available, we provide short historical perspectives and discuss the evolution of insecticide delivery methods and application formulations. Definitions and notions historically and currently used for various pesticide application formulations and methods are also included in this review.

## 2. Gas and Vapor Insecticide Application Formulations

Insecticides can be effectively applied and delivered to the target site in the form of gaseous fumigants or vapors. Gases and vapors form a homogenous mixture of freely and sparsely dispersed insecticidal molecules in the air. This feature distinguishes gases and vapors from aerosols, which are formed by small droplets or smoke particles dispersed in the air. The size of a fumigant gas molecule is more than 1000 times smaller than a liquid aerosol droplet [45]. Anoxic or hypoxic atmospheres are formed by gas molecules already present in atmospheric air. Freeman [32] defined fumigants as pesticide gases that can either pass through the cuticle of the insect or enter through the insect’s breathing system. Thoms and Busacca [45] defined fumigants as pesticides that are in a gaseous state upon contact with the target pest. Hagstrum and Phillips [22] described fumigants as gaseous pesticides that are acute toxins with the capacity to rapidly mitigate an infestation, while leaving very little to no detectable chemical residue. Currently, a fumigant is most commonly defined as a synthetic or natural chemical that, at the required temperature and pressure, exists in a gaseous state in sufficient concentrations to be lethal to a target pest [46].

Fumigation may be broadly defined as a set of processes, procedures, and activities associated with the application of toxic pesticide gas to effectively and safely control pests. Thoms and Busacca [45] discriminated between four basic categories of fumigation procedures, namely, (i) soil fumigation, (ii) fumigation for quarantine and preshipment, (iii) commodity fumigation, and (iv) structural fumigation. All types of fumigation are commonly considered hazardous operations [47]. Therefore, much attention is given to the methods of their application in terms of not only efficiency but also occupational and environmental safety. For most types of fumigation, there are national certifications that are required for companies and specialized personnel in many parts of the world.

All types of gases and vapors can more or less intensively enter the internal spaces of cracks and crevices, voids, machines and equipment, and semi-open structural building cavities. However, only certain groups of gaseous insecticides—i.e., “true fumigants” and inert gases—have unique physical properties, enabling them to permeate and penetrate porous solid matter (such as grain or mill/store construction wood [48]) through molecular diffusion. The important prerequisites for gas penetration ability through solid materials are low physical and chemical sorption, small and linear molecules, and a sufficiently high density of molecules per unit of air volume [46,49,50]. Inorganic or organic vapors and gaseous insecticides used for fumigation procedures may be of either synthetic or natural origin.

**Synthetic and inert insecticide gases and vapors**. Synthetic fumigants and inert gases include very diverse chemical compounds, such as carbon disulfide (e.g., bisulfide) (CS_2_), carbonyl sulfide (COS), chloropicrin, ethane dinitrile (EDN), methyl bromide (MB/MeBr/CH_3_Br), aluminum phosphide (AlP), magnesium phosphide (Mg_3_P_2_), phosphine (PH_3_), sulfuryl fluoride (SO_2_F_2_), carbonyl sulfide (CS_2_), sulfur dioxide (SO_2_), carbon tetrachloride (CTC), acrylonitrile (ACN), ethylene dichloride (EDC), ethylene dibromide (EDB), carbon dioxide (CO_2_), carbon monoxide (CO), nitrogen (N_2_), ethyl formate (EF), ethylene oxide (ETOX), hydrogen cyanide (HCN), methyl iodide (MI), methyl isothiocyanate, methyl formate, methyl benzoate (MBe), ozone (O_3_), propylene oxide (PO), nitric oxide (NO), and acetaldehyde [17,32,40,46,51,52,53,54,55,56,57,58,59]. The general toxicity of most of the above-listed fumigants to a wide range of organisms indicates their adverse effects on fundamental life processes at various physiological levels, including cellular processes [45]. Therefore, most synthetic fumigation compounds and inert gases have broad-spectrum pesticide and biocide effects [56,59,60]; these compounds may be not only insecticides but also fungicides and nematicides [61,62]. Synthetic fumigants may be applied as structural or quarantine pesticide treatments of wood [63,64]; as weed seed devitalization treatments; or as quarantine pesticide treatments of cut flowers, fresh fruits, and vegetables [65]. A detailed description of the multiple and versatile uses of the abovementioned fumigation compounds can be found in numerous scientific articles and in several specialized reviews [57] and books [40,45,46,56,59].

**Natural botanical insecticide gases and vapors**. The active compounds of vapors and gases of natural botanical origin belong to several unrelated chemical groups [66] that include monoterpenoids, cyanohydrins and cyanates, sulfur compounds (dimethyl disulfide, diethyl trisulfide, di-*n*-propyl disulfide, allyl disulfide, diallyl trisulfide, allyl thiosulfinates), alkaloids (Z-asarone), and others (methyl salicylate, benzene derivatives, bornyl acetate, terpinolene). However, a completely clear classification line between natural and synthetic fumigants is hard to establish. For example, in India, a bio-generator was suggested to naturally produce hydrogen cyanide (HCN) from natural plant cyanogenic materials such as cassava [67]. Moreover, in some experiments, researchers have attempted to produce seeds containing cyanogenic multilayers for use as protective coatings (biodegradable polylactic acid, amygdalin, or β-glucosidase). Gaseous HCN is released only when protective layers are ruptured by a herbivore attack [68]. Rajendran and Sriranjini [66] and Campolo et al. [69] showed that the array of natural botanical substances (e.g., essential oils) exhibiting insect toxicity in the vapor phase is much wider than that of synthetic substances, but the extent of their current practical use is profoundly narrower. In fact, we were not able to find any published records (from laboratory or even field experiments) describing the penetration potential of any botanical fumigant to kill internally developing pest stages inside seed kernels. For example, Follett et al. [70] found that basil oil fumigation caused high mortality in adult *Sitophilus oryzae* (Linnaeus) (Dryophthoridae) when exposed to an empty container, whereas pest mortality was low and reproduction was not affected when basil oil was placed in the packaged commodity. Therefore, the authors warned that the effectiveness of plant essential oil fumigation should be evaluated under realistic conditions to avoid experimental artefacts and misleading results. The sorption of the active ingredient by a commodity may be an important technological constraint, as demonstrated by Yang et al. [71]: cinnamon oil exhibited high potential for the control of *S. zeamais* adults in the lab-scale bioassay in empty containers (100% mortality within 24 h); but it failed to exhibit strong insecticidal activity when the container was filled with rice (1.3–12% mortality). Rajendran and Sriranjini [66] further noted that there are limited studies on the effects of essential oils on the nutritional quality of food commodities and on the persistence of their residues.

Recently, methyl benzoate (MBe), a volatile ester associated with snapdragon flower odor, was proposed as a so-called green pesticide [71] and a new promising fumigant candidate. Methyl benzoate is considered a food-grade safe compound approved by both the U.S. Food and Drug Administration and the European Union for use as flavoring and as an adjuvant [72]. The insecticidal activity of MBe was documented for important pest species from several taxa, such as mites, [71], ants [73], moths [74], flies, true bugs [75,76] and storage beetles [72]. Morrison et al. [72] documented, under laboratory conditions, that MBe induced high mortality of storage pests *Rhyzoperta dominica* (Fabricius) (Bostrichidae) and *Tribolium castaneum* (Herbst) (Tenebrionidae) in both the absence and presence of food with increasing MBe doses. The authors also found that MBe is a species-specific fumigant since it failed to kill 100% of *Trogoderma variabile* Ballion (Dermestidae) and *Sitophilus zeamais* (Motschulsky) (Dryophtoridae).

### 2.1. Vaporization and Sublimation

#### 2.1.1. Thermal Vaporization

**Figure 2:**
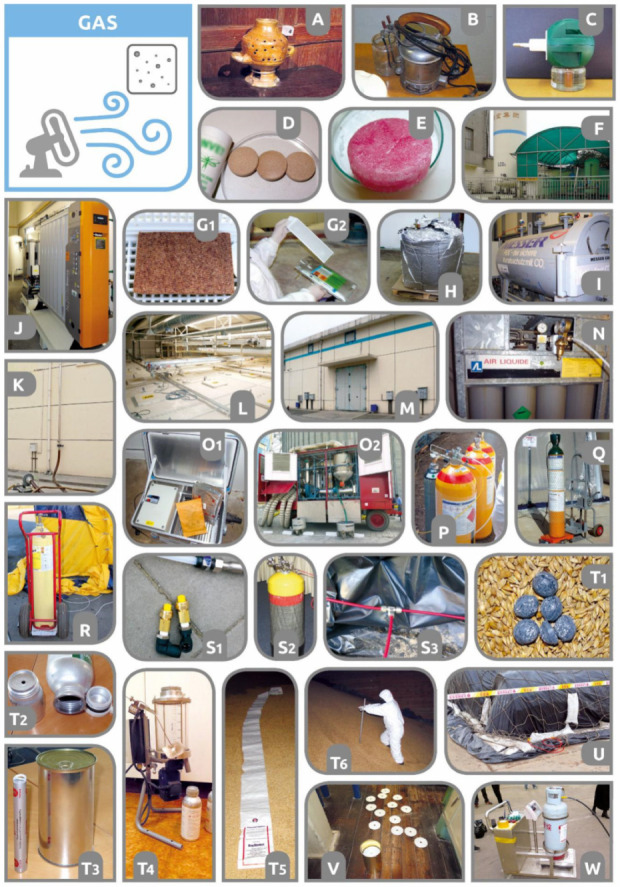
Gases and vapors. (**A**) Historic biocide (disinsection/disinfection) flame heat evaporator; (**B**) historic heat electric evaporator; (**C**) modern electric pyrethroid evaporator for Diptera control; (**D**) example of insecticide (pyrethroids/naphthalene) evaporation tablets (“mothproofers”); (**E**) pressed sublimation block of paradichlorobenzene; (**F**) large CO_2_ tank station for controlled atmospheres; (**G1**) dichlorvos (DDVP) in a porous evaporator matrix; (**G2**) application of DDVP evaporation strips supplied in aluminum packages; (**H**) hypoxic storage bag from a composite foil–modified/controlled atmosphere; (**I**) fumigation chamber—controlled atmosphere; (**J**) nitrogen generator unit—controlled atmospheres; (**K**) fumigation circulation loop—piping with blower (x-ventilator); (**L**) metal vertical silo complex for N_2_ controlled atmospheres in the Czech Republic; (**M**) concrete horizontal storage complex for CO_2_ controlled atmospheres in China; (**N**) cylinders with N_2_ for controlled atmospheres; (**O1**) thermal speed-box for releasing phosphine from magnesium phosphide plates; (**O2**) phosphine gas generator from solid phosphides; (**P**) two cylinders for the coupled release of compressed ethane dinitrile (EDN) gas + gray cylinder with N2 inert propellant; (**Q**) cylinders and piping for releasing compressed phosphine (PH_3_) gas mixed with inert CO_2_ gas; (**R**) cylinders and piping for releasing compressed sulfuryl fluoride (SF) into a freight container sealed by plastic sheets/tarpaulins; (**S1**) spray-nozzle for hydrogen cyanide (HCN) application; (**S2**) cylinders for the release of compressed HCN gas; (**S3**) lines and piping network for the application of EDN and HCN gases; (**T1**–**T3**) solid phosphide tablets for PH_3_ gas release; (**T2**,**T3**) bottle (with inert atmosphere) and traditional can tube-type metal packages for phosphide tablets and pellets; (**T4**) automatic applicator of phosphide round tablets/pellets into grain moving on conveyors; (**T5**) chain of phosphide-containing bags for PH_3_ slow release into a stored commodity; (**T6**) application of PH_3_-generating phosphide tablets into grain mass by a metal hollow spear-probe applicator; (**U**) fumigation under tarpaulin/fumigation sheets; (**V**) release of HCN from liquid HCN-soaked discs after removal from hermetic metal cans; (**W**) quarantine application of compressed methyl bromide (MeBr) from a cylinder container placed on a weight-scale to measure the accurate dosage (photographs (**A**–**W**): V. Stejskal; R. Aulicky, T. Vendl).

One of the oldest ways known to humankind of using pesticides against insect pests and pathogens is the application of vapors or gases using thermal evaporators [77]. In the past, containers of insecticidal aromatic liquids were used, from which insecticidal fumes of natural extracts were released by the heating of candles or oil lamps (Figure 2A). Historically, naphthalene [78], nicotine [79], pyrethrum [80], and rotenone [81] vaporization heaters were developed to control various urban, household, and stored-product pests [82]. The duration of action of these compounds was relatively short due to discontinuous vaporization [83]. In the post-war period, the concept of continuous vaporizers was established mainly in Britain and the USA [83,84]. Lindane (γ-HCH) was used as the main active ingredient [83,85,86]. DDT (dichlorodiphenyltrichloroethane) was also proven to be capable of long-duration continuous vaporization by heat [87]. Although a low DDT vapor pressure led to its condensation into liquid aerosol droplets immediately after vaporization, it also allowed long-term continuous exposure. DDT heat vaporizers were sold primarily for fly control, but they were also suggested for the control of smaller moths and pests of stored goods [87]. Later, these early vaporizers were developed into the new concept of electric biocide (i.e., disinsection or disinfection) vaporizers (Figure 2B). Their advantage was that they were able to ensure the more or less evenly controlled evaporation of biocides and thus simply control pests for several hours or days. We were not able to find any published data on the current use of thermal electric evaporators against storage and food pests at the level of modern warehouses. Modern electric vaporizers, releasing insecticides from containers or soaked porous plates (Figure 2C), are based on pyrethroid preparations and are mainly intended for use against mosquitoes, but there are also products targeted against adult house flies (*Musca domestica* Linnaeus (Diptera)). The application potential of thermal vaporizers can be seen in situations for which they have already been historically used, i.e., for the controlled release of natural botanical volatiles [66]. Research inspiration may be drawn from the published data on tests of botanicals such as neem oil in electric liquid vaporizers [88] or oil kerosene lamps against mosquitoes [89,90]. In the process of developing both new botanical and synthetic heat vapor-based products, the fundamental and practical lessons obtained from the investigation, development, and use of vaporized insecticides since the 1960s should be taken into account. For example, many insecticides are sensitive to decomposition due to heat, and the added alkaline materials and crystalline compounds tend to coalesce into a “cake”, preventing adequate contact between the active ingredient and the thermal resource [87].

#### 2.1.2. Cold Vaporization or Sublimation (“Residual Fumigation”)

In the classical pest-control monograph *Insects and Hygiene*, its main author, J.R. Busvine [91], called the method of the gradual slow release of volatile insecticide substances “residual fumigation” because it provides long-term protection of the treated space by maintaining permanent airborne insecticidal residues. This is why spontaneously released volatile substances (via sublimation or evaporation physical processes) have found widespread use from households to commodity stores and food industry facilities. Wright [92] showed that mercury vapor, slowly released from small kali bottles located in grain, was effective in preventing the reproduction of several species of storage beetles and moths. Mercury vapor was reported not only as an adulticide but also as an ovicide insecticide [93,94]. Beads, tablets, blocks, flakes, and other shaped forms of compressed naphthalene or paradichlorobenzene (PDB) (Figure 2D,E) represent the oldest application formulations used historically for the control of storage dermestid beetles and tineid moths [95,96,97]. From the thin surface layer of such compact forms, insecticide molecules sublimate into the air [98]. Another traditional cold passive vaporization method involves porous cellulosic (paper, fiberboard, etc.) or plastic (resin) plates, strips (Figure 2(G1,G2)), or pellets saturated or impregnated with dichlorvos (DDVP) organophosphates [99,100,101]. Lehnert et al. [102] identified the combination of DDVP vapors with heat stress as a very effective control method. As an alternative to more toxic DDVP, vaporizers were saturated by volatile pyrethroids such as empenthrin or profluthrin [103]. Attempts to use low-volatile pyrethroids were also made, for example, cypermethrin and prallethrin in fabric cotton [104] and esbiothrin in impregnated ropes [105]. The majority of the previously-mentioned active pesticide compounds were extensively used several decades ago. Currently, the number of substances suitable for continuous vaporization purposes is restricted or banned by legislation in a number of countries, and it is difficult to find a specific substitute for these substances. For example, evaporation of DDVP was proven to be very effective for the treatment of cocoa and grain stores [106] against beetle and moth pests [100,101,107,108,109,110]. Continuous evaporation of insecticides was successfully used not only for long-term protection of mills and commodity stores but also to protect warehouses of stored textiles or museum artefacts and collections as “mothproofers” [111,112]. Linnie and Keatinge [113] identified the application of DDVP as the most effective (particularly against dermestid larvae and adults), followed by paradichlorobenzene, whereas naphthalene was the least effective. As a potential alternative to synthetic pesticides for passive cold vaporization, the use of volatile botanicals of natural origin has been suggested [66,114]. Unfortunately, this research remains mainly in the stage of laboratory experiments and lacks the convincing field validations required for their adoption for broad industrial use.

#### 2.1.3. Preventive vs. Repressive Application Methods of Insecticide Evaporators

Most evaporators are typically applied preventively to reduce damage risks and provide long-term protection [106]. For example, DDVP evaporation strips are hung on ropes and cords above grain or bags in a closed area of a mill or a flat store [107,109]. Peters [107] stated that DDVP strips should be preventively applied before moths begin to emerge in the spring and that the exposure period should last four months. Bengston [115] estimated the time of daily emission of DDVP needed for the control of the almond moth *Ephestia cautella* (Walker) (Lepidoptera). To ensure the effectiveness of such emission rates in practice, the treated space should be enclosed and lack ventilation because air exchange reduces the vapor concentration. However, the absence of ventilation may lead to the unwanted exposure of personnel. Therefore, Aulicky et al. [116] tested two DDVP evaporation regimes with strips, namely, “preventive” and “repressive”. In the “preventive” regime, the strips were introduced 168 h before pest exposure, whereas in the “repressive” regime, strips were introduced concurrently with pests. The data showed that short-term exposure to DDVP strips has a suppressive effect on *Oryzaephilus surinamensis* (Linnaeus) (Silvanidae) but cannot fully replace long-term exposure of strips or high-dose DDVP aerosols for other tested species of stored pests (*T. castaneum*, *Cryptolestes ferrugineus* Stephens (Laemophloeidae), *R. dominica*, and *Sitophilus granarius* (Linnaeus) (Dryophthoridae).

#### 2.1.4. Injection/Infusion into and Evaporation Inside Bags (“In-Bag Fumigation”)

The method of fumigation of individual sacks [117] using the injection [118] of vaporizing volatile liquids into packages was established almost 50 years ago and was termed “in-bag fumigation” [119] or “individual package fumigation” [52]. For this purpose, fumigants such as DDVP [118], carbon tetrachloride [120], methallyl chloride [117], and chloropicrin [121] were proposed. Green and Wilkin [118] described an injection method based on DDVP dissolved in carbon tetrachloride and dispersed it into the air stream passing from a motorized knapsack sprayer to a perforated lance, which was pushed into the grain. Using this method, DDVP was distributed evenly through the intergranular spaces of bagged wheat and barley and provided a good level of control for *O. surinamensis* and *S. granarius* [118]. Monro [52] reported that acrylonitrile (ACN), carbon tetrachloride (CTC), or ethylene dibromide (EDB) liquid fumigants could be injected or inserted (as soaked porous discs in aluminum foil) into double walled polyethylene or jute bags filled with the treated commodity. Recently, Tola et al. [122] suggested that plastic hermetic storage enclosures can be combined with the infusion of smoke to cause anoxia.

### 2.2. Fumigation—Toxic Gas Release from Solid, Liquid, and Gas Formulations

Fumigation is one of the most ancient methods of pest control. However, the foundations of modern fumigation were not established until the first three decades of the 20th century [52]. The goal of fumigation is to deliver and maintain a sufficient concentration of a gaseous fumigant long enough to kill all stages of the target species. For fixed environmental conditions, the effective pesticide dosage can be described by a function of the fumigant concentration and the fumigation exposure time, usually expressed as various mathematical forms of the so-called concentration (C)-time (t) product (Ct-P) [46]. Thoms and Busacca [45] explained that the target Ct-P-based dosage could be achieved by varying either concentration (C) or time (t) to produce a toxic effect on the target pest species. Ct-P is specific not only for a particular pest species but also for its particular developmental stage [123]. For example, eggs and pupae of stored-product Coleoptera usually require higher phosphine doses and Ct-P than larvae or adults [124]. Insecticidal formulations of gases and vapors have a high potential for relatively rapid spatial distribution in warehouses and buildings of food operations through molecular diffusion or via convection or advection air currents [50,125,126]. Due to sorption, both synthetic fumigants [126,127] and natural compounds may not always be effective enough to enable the even spatial distribution of gases [70].

Traditionally, fumigant formulations are categorized as “solid”, “liquid”, and “gas” [52,107,128,129,130]. This classification is practically important because it describes not only the methods of transport of fumigants but also the methods of their application. Depending on the needs of a particular application, it may be technologically advantageous to choose whether an identical active ingredient (e.g., phosphine) will be released from a gaseous (cylinderized) or solid formulation (tablets, pellets, etc.).

#### 2.2.1. Fumigants Released from Solid Formulations

Fumigants may be released from various types of solid formulations that include either pyrotechnic preparations or chemically reactive solid formulations [33,52]. Pyrotechnic fumigation preparations are quick-release gas formulations that mainly include cartridges, sulfur wicks, or candles. Combustion of pyrotechnic fumigation cartridges containing sodium nitrate (NaNO_3_) and charcoal-carbon (C) produces toxic carbon monoxide (CO), along with gaseous nitrogen (N_2_) and solid sodium carbonate (Na_2_CO_3_) [131]. Such cartridges are mainly used for the control of pest vertebrates in burrows [132]. Sulfur produces sulfur dioxide (SO_2_) after the ignition [51] of a wick inserted in a metal can filled with sulfur waxed pellets and additives. Among all fumigants, sulfur is the most ancient [52]. Moreover, a sulfur candle was the first patented fumigation formulation (applied in 1897 in the USA: US66129597A). Solid reactive preparations are slow-release formulations from which the gaseous active ingredients are mostly released by chemical reactions with water, moisture, or an acidic environment. The distant insertion (using ropes/strings) of bags with solid cyanide salts into barrels with acid liquids is known as the “stringing method of HCN fumigations” [133]. Currently, the most commonly used solid compounds are metal phosphides (aluminum/magnesium phosphide), which gradually release gaseous phosphine (PH_3_) after reacting with H_2_O vapor in air [46,134,135,136]. Similarly, in the past, solid calcium cyanide, Ca(CN)_2_, was frequently used to release gaseous HCN through the reaction of Ca(CN)_2_ with air humidity [52]. The application of finely divided calcium cyanide salts was historically known as “dust fumigation” [17]. Wardle [17] described a special solid–liquid hybrid formulation of HCN (e.g., Citrofume [137]) as a “*fine powder formed by liquified hydrocyanic acid gas with calcium carbide, which is practically pure calcium cyanide and contains 30% hydrocyanic acid*”. Commercial solid reactive preparations include dust, powder, crystals, or various compressed forms (tablets (Figure 2(T1)), pellets, granules, etc.). Until the beginning of a fumigation process, the compressed phosphide formulations were enclosed in hermetic protective metal cans (Figure 2(T3)) or flasks/bottles filled with protective inert gases (Figure 2(T2)). Compressed phosphide formulations can be formulated in so-called prepacked ropes or pouches enclosed in metal transport containers. Solid fumigants can also be applied in packaged (paper; diffusion foils, e.g., Tyvek^®^; etc.) formulations, including bags, satchels, sachets, blankets, chains (Figure 2(T5)), strips, and plates [138]. If legally allowed, tablets and pellets may be filled and confined into a gas-permeable paper envelope or textile bags and sleeves [139]. Some packaged application formulations generally decrease the speed of the gas release [140] but protect the treated space, commodity, and workers from direct contact with fumigation dust residues. In the literature, we did not find any example of industrially used solid fumigants of natural botanical origin.

Solid synthetic fumigants include cyanide salts [46,52] and widely used metal phosphides (used along with ammonium carbamate, zeolites, etc.) [139,141]. The application of solid fumigants is carried out either in the spaces of structures [54] or directly as an admixture in a static or moving commodity [138]. Solid pressed formulations of fumigants are located on trays near the material or pallets with commodities to be fumigated [139]. Phosphides may be delivered into a static commodity in the form of tablets, bags, and chains for surface or subsurface applications. Deeper delivery of tableted or pelleted phosphide formulations into static grain mass is realized via their application through hollow tube spears (Figure 2(T6)); phosphide spear-applicators may be naked or covered with textile sleeves. The most effective and even distribution of phosphine in grain mass (e.g., silos, ships, flat-horizontal stores, containers) is achieved through pre-installed tubing or loops equipped with forced gas circulation using blowers/ventilators [142]. Partial treatment of stored grain, known as “spot fumigation” [52], may result in low efficacy [143]. Direct mixing with a moving commodity (i.e., fumigant application in a grain stream during filling or transfer from one bin to another) allows the fumigant to be applied and distributed evenly. Aluminum/magnesium phosphide tablets or pellets (or calcium cyanide dust/crystals in the past) can be applied manually in the grain stream or discharged with a semi-automatic dispenser (Figure 2(T4)). To increase the speed of the phosphine release from solid formulations, either chemical reaction-based generators (Figure 2(O2)) [144,145,146] or heat-based accelerators have been constructed [147] (e.g., Figure 2(O1)—Degesch SpeedBox for magnesium phosphide plates). Waterford and Asher [148] classified phosphine generators as rapid-release (rate of release > 1 kg of PH_3_ per h) or slow-release (rate of release ≈ 4–8 g of PH_3_ per h) devices. Grain store loop fumigant recirculation techniques can include a combination of both generator types: a quick release to rapidly establish and distribute the fumigant and a slow release to maintain the target concentration for the remainder of the fumigation [148]. Formato et al. [149] proposed a new device to accelerate the reaction between phosphide pellets with air moisture based on a heated cylindrical tray and the remixing of pellets subjected to contemporaneous nebulized water sprinkling.

It should be stressed here that there are a vast number of published studies on the various phosphine application technologies, the coverage of which is far beyond the scope of this review. For example, there was a recently published extensive paper [142] showing phosphine concentration dynamics using various application formulations and technologies (phosphine forced circulation) under diverse commodity storage or transport conditions (stores, ships, freight containers) in Greece.

#### 2.2.2. Fumigants Released from Liquid or Liquidized Gas Formulations (Compressed in Cylinders, Soaked in Porous Materials)

Physical formulations of fumigants are classified, apart from solids, as liquids or gases. This fact may be slightly confusing since virtually no, or very rarely any, fumigation preparation occurs in gaseous form when stored or transported in cylinders or cans. The only “gas-from-gas” release exception is ozone (O_3_), which is produced with an ozone generator (e.g., molecular oxygen is transformed into ozone by means of an electric charge) that acquires oxygen directly from the ambient gaseous air [55]. A fraction of liquidized phosphine gas may also appear at the top of the metal pressure cylinder with liquidized nitrogen. For example, the application manual for the VAPORPH3OS^®^ Phosphine Fumigant states that “*The product flows to the blending equipment through the cylinder outlet valve. As gas is withdrawn from the cylinder, some of the product vaporizes to fill the remaining space in the cylinder. Through this vaporization, the cylinder pressure is maintained.”* (http://www.fumigationzone.com/files/ac/VAPORPH3OS-Manual2013-English.pdf (accessed on 24 June 2021)).

Traditionally, the term “liquid fumigant” (i.e., “low-pressure fumigant”) has been suggested [32,52,128,129,130] for groups of volatile fumigants (e.g., carbon tetrachloride, ethylene dichloride or dibromide) of which the boiling point is sufficiently high for them to be liquid at normal atmospheric temperatures and pressures in enclosed containers. These fumigants are considered to be distinct from “gas fumigants” (i.e., “high-pressure fumigants”) (e.g., methyl bromide, EDN), which are gaseous under these conditions, and in order to be liquids, they have to be compressed and stored in steel cylinders [128]. Historically, liquid fumigants were simply poured or sprinkled from bottles/containers directly onto packages, bagged commodities, or the floor of the treated structure [32,128]. Carbon tetrachloride was even tested for application as a thermal aerosol in combination with DDT [150]. Some types of liquid low-pressure fumigants (acrylonitrile, vinyl cyanide, trichloroacetonitrile) were injected into the grain bulk mass using hydraulic injector spears with motorized pressure generators [130]. These fumigants were also applied through the soaking of porous discoids [151] or hung in the space on ropes [46,52,152]. Small cardboard discs impregnated with ethylene dibromide (EDB) were wrapped in foil sachets for fumigation of a small unit of grain; each sachet was cut open immediately before disk insertion into the grain mass [52]. Liquid fumigant structural fumigations were even performed manually, using pressured sprayers [52]. In the past, low-pressure fumigants and their mixtures were commonly used for empty grain stores and commodity fumigation [32,52,153], space flour mill treatment, and notably for “spot treatment” of certain machinery (covered by plastic sheets) in flour mills [129]. Later, Quinlan and Gaughey [154] tested the activity of chloropicrin, phosphine, and liquid fumigant mixtures of carbon tetrachloride and carbon disulfide for fumigation-infested grain dockage in empty grain bins. More recently, a mixture of ethyl formate and methyl isothiocyanate was suggested for grain treatment [155], and ethyl formate alone was proposed as a grain surface and empty silo treatment [156]. Although the results of recent studies are promising, few low-pressure liquid fumigants are currently registered or broadly used worldwide.

Liquidized gas fumigation preparations, such as hydrogen cyanide (HCN), may be distributed as stabilized liquid-soaked porous materials (discoids, chips, granules, etc.) enclosed in metal cans (0.5–1.5 kg) (e.g., [46,151]). HCN application is performed by opening cans with a metal opener and spreading porous discoids on the floor (Figure 2V), from which the gas is gradually and autonomously released into the treated space. More commonly used gases are compressed in cylinders or cans constructed for stabilized toxic liquids. Examples of liquidized fumigants supplied in cylinders may include phosphine (Figure 2Q), methyl bromide (Figure 2W), hydrogen cyanide (Figure 2(S2)), sulfuryl fluoride (Figure 2R), ethane dinitrile (EDN) (Figure 2P), carbon dioxide, propylene oxide, and ethyl formate [59]. Phosphine, supplied in compressed cylinders, has two basic formulations and concentrations [136,157]. The highly diluted phosphine formulation in CO_2_ can be applied directly (Figure 2Q). Non-flammable gaseous phosphine mixtures with inert gases (i.e., low concentration of PH_3_—usually below 1.8%–2%—diluted in a high concentration of N_2_ or CO_2_) may eliminate the PH_3_ flammability hazard [158,159]. The use of a highly concentrated (>99%) formulation requires special equipment for “on-site” blending of phosphine with insert gas (e.g., carbon dioxide) and forced air [160]. Similarly, due to ethyl formate (EF) flammability, EF is mixed with carbon dioxide in pressurized cylinders (e.g., 16.7% EF dissolved in CO_2_ [161]). To reduce the flammability of propylene oxide (PPO), this gas is diluted by an inert gas propellant (2% PPO + 98% CO_2_) [59].

Compressed gas fumigants may be applied directly by placing cylinders in the fumigated space and opening the release valves. Distant and thus safer application is ensured by using thick-walled tubing networks (Figure 2(S3)) introduced into the treated space (e.g., grain stores, mills, sealed freight containers (Figure 2R)—where cylinders and the service fumigation staff remain outside the fumigated space. Thick-walled tubing/pipelines may be permanently pre-installed in regularly fumigated structures such as flour mills. Pipelines must be pressurized with propellant to force fumigants into multi-store buildings. Liquidized fumigants may be discharged into a fumigated space through special nozzles (e.g., HCN—Figure 2(S1)) or through a shallow evaporating pan (e.g., methyl bromide). However, for the application of large volumes of methyl bromide, such as during ship quarantine fumigation, heat exchanger stations or mobile units are required for effective and rapid evaporation; this is called the “hot gas” fumigation method [52]. Some fumigants require active ventilation (insulated combustion-safe X-fans) for their quick dispersion in space (Figure 2K). For the application of phosphine in silos, vertical forced circulation is recommended. For the application of “heavy” and sorptive fumigants, powerful (“forced”) circulation is required. Apart from the abovementioned methods, many other application techniques and technologies are used for structural fumigation (mostly for flour mill treatment), chamber fumigation (normal/changed pressure and temperatures), and bagged commodity fumigation (stores, ships, freight container tents, bubbles, under sheets/tarpaulins (Figure 2U)). However, a detailed overview of these is beyond the scope of this work, though it is available in other original works, reviews, and monographs [40,46,52,59,142,162].

### 2.3. Application of Inert Gases as Modified Atmospheres (i.e., Pest Asphyxiation by Hypoxic/Anoxic Atmospheres)

Although simple inert gas technologies date back to the early stages of human agriculture [77,163], the possibility of asphyxiating insects affecting industrially stored food in hermetically sealed enclosures and containers was suggested in 1918–1922 in the UK and Australia [164,165,166,167]. Hypoxic or anoxic technologies are based on atmospheric gases, but their concentrations are manipulated and changed. According to Navarro [168], the terminology and classification for hypoxic and anoxic atmospheres are not used uniformly. He suggested that “modified atmosphere” (MA) represents the most general term for any type of hypoxic and anoxic atmosphere. MAs may further include: (i) controlled atmospheres (CAs) and (ii) hermetic or airtight storage (i.e., “sealed storage,” “airtight storage,” or “sacrificial sealed storage”). Both types of treatments may occur under normal or altered atmospheric pressure. Hermetic or airtight storage is based on naturally modified atmospheres (with an increased content of CO_2_), gradually created by natural breathing of stored grain and grain-associated microorganisms and pest arthropods. The conditions for good and long-term efficacy of hermetic commodity storage are properly sealed storage construction, preventing the re-entrance of oxygen from the surrounding air atmosphere [56,169], as well as protection against gnawing activity by rodents. Navarro [168] suggested “assisted hermetic storage” as a special subcategory of hermetic storage. This approach is based on burned biomass, exothermic gas generators, catalytic oxygen converters, or respiration gases of plant material [122,170,171]. Even in this case, the atmosphere is modified by the supply of gases generated outside the hermetic storage enclosure. Controlled atmospheres are based on the fully controlled introduction of externally supplied inert gases into hermetic enclosures to reach and maintain the target concentration and exposure. The modified gas composition is produced artificially [168]. For example, Mohammed [172] demonstrated an automated system for pest management on stored dates using a controlled atmosphere approach. The current atmosphere of the Earth contains (by volume) 78.09% nitrogen (N_2_), 20.95% oxygen (O_2_), 0.93% argon (Ar), 0.036% carbon dioxide (CO_2_), 0.0005% helium (He), and other trace gases [173]. Ozone (O_3_) is also naturally present in the atmosphere surrounding the Earth [57]. Concentrations of the most abundant inert gases such as N_2_ and CO_2_ may be increased at acceptable costs, or reactive oxygen (O_2_) can be used at reduced levels [174]. Helium may be effective as an insecticide, but it is too costly. Technologies involving anoxic atmospheres are environmentally friendly, as they do not leave chemical residues in the treated commodities and do not deplete the ozonosphere like methyl bromide. Resistance to inert gases has already been reported [175,176,177,178] but this is not even at a level comparable to that documented for some fumigants or conventional neurotoxic sprays or dusts. Therefore, inert gases are suitable substances for the protection of sensitive high added-value commodities or sensitive stored products, especially in the case of organic farming or baby-food production.

#### 2.3.1. Hermetic Airtight Storage (Bags, Cocoons, Bunkers, Underground Stores and Pits, Under-Sheets)

Underground storage with hypoxic/anoxic atmospheres represents one of the oldest methods used in early agriculture until the Middle Ages [56,179]. Such simple stores are still conserved at historical archaeological sites or are still in operation in some rural areas of developing countries [163,180]. The background of modern airtight storage was established in the 1950s (e.g., [181,182]). The currently used sealed structures for hermetic/airtight storage include a variety of constructions and technologies, e.g., ferro-cement concrete stores [183], pits and bunkers covered with plastic sheets (storage capacity 10,000 to 20,000 t), PVC tanks [184,185], and numerous portable or static flexible containers and structures of variable size (e.g., 60-kg to 2-ton SuperGrainbagsᵀᴹ; 1-ton-capacity Grainsafe IIᵀᴹ; 5- to 1000-ton capacity cubes or Cocoonsᵀᴹ) [186]. In Argentina, an original system of hermetic big grain plastic bags was developed [187] to serve as a temporary storage structure for freshly harvested grain before its transport to permanent stores. Most likely, one of the most prospective airtight bag-type containers for large-scale adoption in practice [30] is relatively small (~50 kg) hermetic multilayer plastic storage bags primarily designed for smallholder farm storage [188]. The first pioneers of hermetic commodity storage using such small bags were Wilkin and Green [189] from the former Central Science Laboratory (MAFF), Slough, UK. They tested the efficacy of bags with polythene outer sacks of 0.127-mm wall thickness and found that commodities infested with *O. surinamensis* and *S. granarius* created (due to respiration) an atmosphere of 14% CO_2_ and killed the tested pests in three days. With the usage of new-generation materials, multilayer (e.g., double plastic bags [190] and triple-layer bagging [191], i.e., “triple bagging” [29]) bags were developed that ensure the generation and maintenance of hypoxic modified atmospheres. The outer layer of some products incorporates pyrethroid insecticides [192]. Efficacy tests are available for multiple species and commodities from different countries and environmental conditions [188,193]. Recently, Ngwenyama et al. [194] compared the efficacy of five hermetic bag brands (GrainPro Super Grain bags (SGBs) from IVR™; PICS bags; AgroZ^®^ Ordinary bags; AgroZ^®^ Plus bags; and ZeroFly^®^ hermetic bags) with dust with pirimiphos-methyl and found almost identical performance. Due to the high efficacy, low cost, and simple operation of these bags, the potential users are mainly small and medium-sized farms in developing countries. A drawback of hermetic bags is that the larger grain borer *Prostephanus truncatus* (Horn) (Bostrichidae) [195], *R. dominica* [196], and rodents [194] can perforate the plastic liner of some types of bags, which increases the oxygen levels and negatively affects the efficacy of the method. In contrary, Otitodun et al. [197] claimed that the ZeroFly^®^ bags were not easily penetrated by stored-product insect pests under field conditions.

#### 2.3.2. Controlled Atmospheres in Food Packages, Chambers, Silos, Horizontal Stores, and Flexible Enclosures (Normal/Changed Atmospheric Pressure)

Controlled inert atmospheres (i.e., the artificial introduction of N_2_ or CO_2_ and/or the removal of O_2_ by means of a vacuum or the insertion of satchels with absorbers of O_2_) are most frequently used in hermetic food packaging by food industry producers to protect food products against pests, spoilage, and loss of quality [198,199,200]. The second most common application of inert gases in the form of controlled atmospheres is for the purging of hermetic metal chambers (Figure 2I). Such chambers enable effective manipulation of the atmospheric composition since they easily maintain low O_2_ concentrations and allow changes in pressure and temperature [163]. Increased or reduced pressure and increased temperature substantially reduce the time required for effective (lethal) exposure of all pest developmental stages. Freight and sealed containers may be adapted to fumigation chambers. A flexible chamber/bubble for controlled atmospheres for organic product storage was constructed in the Czech Republic, in which oxygen-proof penetration flexible plastic liners and metal-composite materials were used (Figure 2H). The chamber may be saturated by N_2_ or CO_2_ from a cylinder or a small portable N_2_ generator. Controlled atmospheres may be achieved in adapted and sealed silos [201,202,203] or horizontal flat stores (Figure 2M). Small objects can be treated using multiple cylinders (Figure 2N), whereas effective purging of inert gases into larger objects requires large gas containers and tanks (with pressure relief valves and chambers; e.g., Figure 2F—CO_2_ tank) or gas generators (Figure 2J). Gaseous N_2_ may be obtained through low-cost on-site production from the atmosphere, e.g., by using adsorption generators (fixing various gas mixture components using a solid adsorbent substance) or by using pressure swing adsorption (PSA) technology that employs a carbon molecular sieve [56]. Large tanks or generators allow the creation of overpressure in incompletely hermetic horizontal stores and vertical silos (Figure 2L) [201].

## 3. Delivery of Insecticides as Liquids (Admixtures, Liquid Baits, Aerosols, Sprays, etc.)

### 3.1. Grain Protectants—Spray, Drip, Cob-Dip, and Aerosol Treatments (Insecticide Admixture, Top-Dressing)

In warm geographical areas and some storage environments, stored commodities cannot be efficiently cooled to temperatures that ensure safe storage [56,204]. As an IPM alternative [205], direct treatment of commodities (cereal grains and legumes) with insecticide and acaricide protectants has been suggested [206,207,208,209]. Grain protectants are most commonly applied preventively as grain mass is loaded into storage grain. These compounds are expected to provide long-term residual protection of the treated commodity against a broad spectrum of arthropod pest species over a period of several months [40,209].

Currently, there is a limited number of registered grain protectants [40]. However, over the course of history, there have been a profound variety of active ingredients and application formulations (dust, slurries, or liquid sprays) used as grain protectants. In the past, even metal-based chemicals such as pure mercury, zinc, or tin amalgam were tested and suggested as grain protectants [210]. Shepard [211] claimed that seed corn used to be protected from insect injury by dipping the ears in oil emulsions (diluted one part to 10 parts of water) and miscible oils such as those used for spraying fruit trees. Organochlorines (e.g., DDT and γ-HCH, i.e., lindane) were applied mainly as solid dust admixtures [32,212] or slurries (DDT + inert pyrophyllite) [208]. Pyrethrins synergized with piperonyl butoxide (PBO) have been used as dusts, slurries, or water sprays from emulsified concentrates [208]. Organophosphates such as malathion and DDVP were among the first synthetic organic chemicals widely applied as spray insecticide protectants [206]. Some volatile organophosphate substances have been documented to exhibit certain fumigation effects [213]. However, the usage and registration of various carbamates (carbaryl) and organophosphates (e.g., malathion, dichlorvos, pirimiphos-methyl, chlorpyrifos methyl, diazinon, fenitrothion, fenthion) has been in decline over the last several decades. At present, the remaining pyrethroids (deltamethrin, cypermethrin, and bifenthrin, eventually mixed with piperonyl butoxide—PBO—as a synergist) are the neurotoxic insecticide active compounds most commonly used as grain protectant formulations [209,214]. The prevalent reliance on pyrethroid protectants may result in decreased sensitivity or resistance of storage arthropods [215] as already found in some pest species, populations, and geographical areas [40,216]. The new generations of neurotoxic (neonicotinoids, phenyl-pyrazoles, pyrazoline-type oxadiazines, anthranilic diamides) or ATP-disrupting (halogenated pyrroles) compounds tested as grain protectants have included chlorfenapyr, indoxacarb, ethiprole, fipronil, imidacloprid, thiamethoxam, and chlorantraniliprole [217,218]. In 2018, Daglish et al. [40] stated that, in general, all newly explored compounds showed potential in the laboratory at varying doses depending on the species tested, but none has progressed to extensive field trials or even a registration stage. As reduced risk/low-risk insecticides, formulated as spray protectants, some researchers have considered insect growth regulators/disruptors (IRSs/IGDs) and spinosyn microbial insecticides (spinosad [219,220] or spinetoram [221]), as well as botanicals. Various botanical extracts and essential oils have been tested as grain protectants [222]. For example, Athanassiou et al. [223] found that azadirachtin (neem seed oil solution) was very effective in controlling three Coleoptera pest species, *S. oryzae, R. dominica,* and *T. confusum*. However, the effective dose rates were much higher than those of the currently used grain protectants, thus constituting an unrealistic application in practice.

Recently, Kavallieratos et al. [224] tested an essential botanical oil-based nanoemulsion (HvNE) (isolated from *Hazomalania voyronii* (Jum.) (Hernandiaceae)) applied as a wheat grain protectant against three storage Coleoptera species. After 7 days following exposure to HvNE at a concentration of 1000 ppm, the mortality of *T. confusum*, *T. castaneum*, and *Tenebrio molitor* Linnaeus (Tenebrionidae) adults reached 92%, 97%, and 100%, respectively. However, despite some promising laboratory results, with the exception of natural pyrethrum, botanical insecticides still do not belong among the internationally recognized industrial grain protectants [69]. As mentioned above, one of the reasons is that botanicals, applied as grain protectants, generally require a high dose/application rate [222,223]. Currently, it is likely not practical to consider using botanical grain admixtures in large industrial grain stores because of the fairly large amounts of plant material/essential oil extracts required for such treatments [222]. Weaver and Subramanyam [222] gave the following illustrative example: “*Assume that treatment with 100 parts per million of an extract is required to protect a given commodity. To treat 1000 metric tons of a commodity one would require 100 kg of the extract. If one assumes a 5% yield of extract, which is reasonable, then one would require about 2 metric tons of the raw plant material*.” Nevertheless, vegetable oils and other botanical liquid extracts may have certain local importance as home-made liquid seed coatings and protectants [225,226].

A detailed overview of various formulations and active compounds used as protectants and their effectiveness on various pests and commodities has been described in several reviews [35,40,209,212]. From these reviews and other published works, it is clear that for the practical selection of a particular active ingredient and formulation of a grain protectant, specific conditions should be considered for each particular store and pest species. The conditions affecting grain protection efficacy and cost-effectiveness [227] may include multiple factors [212], such as the type of commodity, the length and method of storage, pest species (e.g., bostrichid beetles are more tolerant to organophosphates than to pyrethroids [228]), various levels of resistance among pest populations [229], the temperature and humidity of the commodity [230], and the current level of or customer-required “maximum pesticide residue level” (MRL). The MRL values may be accidentally exceeded even in non-treated commodities. This issue may be caused by chemical cross-contamination when the untreated commodity is consequently transported by transport routes identical to those of the insecticide-treated commodity [231].

**Insect growth regulators/disruptors (IGRs/IGDs) as grain protectants.** Insect growth regulators (IGRs) or insect growth disruptors (IGDs) [232,233] are compounds that disrupt the life cycle of an insect, mainly interfering with the normal embryonic development, molting, egg-hatching, and cuticle formation processes. They are thus primarily targeted at juvenile insect stages, for which some of these effects are gradually lethal. However, they may also negatively affect pest adults; e.g., in terms of fertility, behavior, pheromone production, etc. [234,235]. IGRs/IGDs are commonly classified into three groups that include analogues of juvenile hormones (juvenile hormone agonists (JHAs)), chitin synthesis inhibitors (CSIs), or ecdysone inhibitors (ecdysteroide antagonists) (EIs/EAs) [233,234,236]. Although many IGRs/IGDs are called “reduced risk”, “low-risk”, or “biorational pesticides” [237], their registration is subject to analogical or identical procedures as those of the remaining insecticide groups in many countries (e.g., in the EU). Various types of IGRs/IGDs show differential activity on various pests. For example, in an extensive laboratory study, Kavallieratos et al. [236] compared the efficacy of a broad variety of IGRs/IGDs as wheat grain protectants against *P. truncatus* and *R. dominica*. Their tests included two JHAs (fenoxycarb and pyriproxyfen), four CSIs (diflubenzuron, flufenoxuron, lufenuron, and triflumuron), and one ecdysteroid antagonist (methoxyfenozide). Although many IGRs/IDGs and their combinations have been experimentally tested, only a few have been introduced for stored grain protection [40]. Among IGRs/IGDs, methoprene (r/s-methoprene, a mixture of r- and s- enantiomers) and s-methoprene (s- enantiomer) are the few compounds that have not only been tested but have also reached the registration and commercial usage stages [234,238,239]. As grain protectants, r/s methoprene (USA—1980s) and s-methoprene (USA—2002) were first registered in the USA and Australia [214,238,239]. The biological efficacy of r/s methoprene and s-methoprene as protectants of various commodities against storage pests has been evaluated by several authors [214,235,240,241,242]. For example, Arthur [240] tested the efficacy of methoprene on *R. dominica* for multi-year protection. He applied methoprene as a stand-alone application (1.25 and 2.5 ppm) on stored hard red winter wheat, brown rice, rough rice, and corn. Methoprene resulted in residual control of stored product beetles for 24 months. Later, Arthur [214] demonstrated that the tested spray grain protectant containing methoprene and/or deltamethrin showed an insecticidal effect on *T. castaneum* and *R. dominica* for 15 months, when applied to corn kernels, and methoprene grain treatments can be effectively combined with low temperature controlled aeration to manage insects in stored wheat [243].

**Spray or aerosol grain admixture.** All types of liquid protectants are applied in such a way that the surface of individual grain kernels or maize cobs is covered/impregnated by a thin layer of insecticide. Although some publications classify these protectants into the category of contact insecticides [244], in practice, they act primarily (major effect) as oral and secondary (minor effect) as respiratory poisons [213]. Therefore, from the pest perspective, the treated grain in a store virtually acts as a “huge bulk of a toxic bait”. Pest intoxication occurs when an adult or larva chews (out or in) through the thin insecticidal layer on the surface of the treated kernel. Oral intake of the insecticide and its entry into the digestive tract of the arthropod body allows the use of very low doses of active ingredients and thus ensures an acceptable insecticide maximum residue level (MRL) in the treated commodities. In most cases, treatment with protectants is carried out during harvest as well as before and during storage. It should be emphasized that the “seed dressing and coat” kernel treatment category is different from the usage of “grain protectants” [36,217]. For seed dressings and single or multilayer coatings, different registered active substances and application devices are used, and warning seed coloration is required [36]. For seed coating/dressings, 100% coverage of all individual seed kernels is required, and seed companies usually perform the treatment. In contrast, grain protectants are colorless and are applied by farmers or storekeepers. Unusually, only a portion of the seeds from the commodity volume are treated [40]. In the scientific literature, there is no general agreement on whether partial grain spray treatment can lead to acceptable efficacy on storage arthropods. It seems that the efficacy of a partial treatment is condition-dependent and is influenced by the extent of grain coverage, pesticide compounds and formulation, species of storage pests, and the sensitivity (resistance/tolerance) of the particular population [212,245]. For instance, Subramanyam et al. [246] claimed that complete control of *R. dominica* adults can be achieved if more than 50% of the kernels receive spinosad treatment. Daglish and Nayak [238] warned that uneven application may reduce the efficacy of s-methoprene in non-susceptible *R. dominica* populations. The results obtained for a deltamethrin protectant showed that long exposure times and treatment of an entire rice mass may be necessary to give complete control of the beetles *T. castaneum* and *T. variabile* [247]. Arthur [214] found that the partial treatment of a grain mass using deltamethrin (EC) did not give optimum control of either sensitive *R. dominica* or more tolerant *Sitotroga cerealella* (Olivier) (Gelechiidae). Under some of the experimental conditions, where 100% brown rice was treated, nearly complete control was observed for *R. dominica,* whereas only a 35% reduction in *S. cerealella* progeny production was observed [214]. Similarly, Scully et al. [248] observed that *R. dominica* was more susceptible, as mortality and knockdown were observed in mixtures containing 10% brown rice treated with Storicide II, whereas *S. cerealella* was less susceptible, as mixtures containing at least 50–75% of treated brown rice were required to reduce progeny production. However, even if the commodity is partially treated, it is still necessary to ensure the relatively even application and admixture of the insecticide throughout the volume of the commodity. The reason for this is, as explained by Daglish et al. [40], that uneven distribution of pesticide protectants may lead to the occurrence of zones within the grain bulks that are under-dosed or even untreated areas, which allow insect colonization and progeny production. The necessity of even protectant distribution is not only to ensure biological efficacy against pests [238,246] but also to prevent local exceedances of insecticide maximum residue levels (MRLs). To reduce the risk of residue accumulation, it has even been suggested to incorporate insecticides such as chlorpyrifos-methyl in a xanthan gum biopolymer [249].

**Figure 3:**
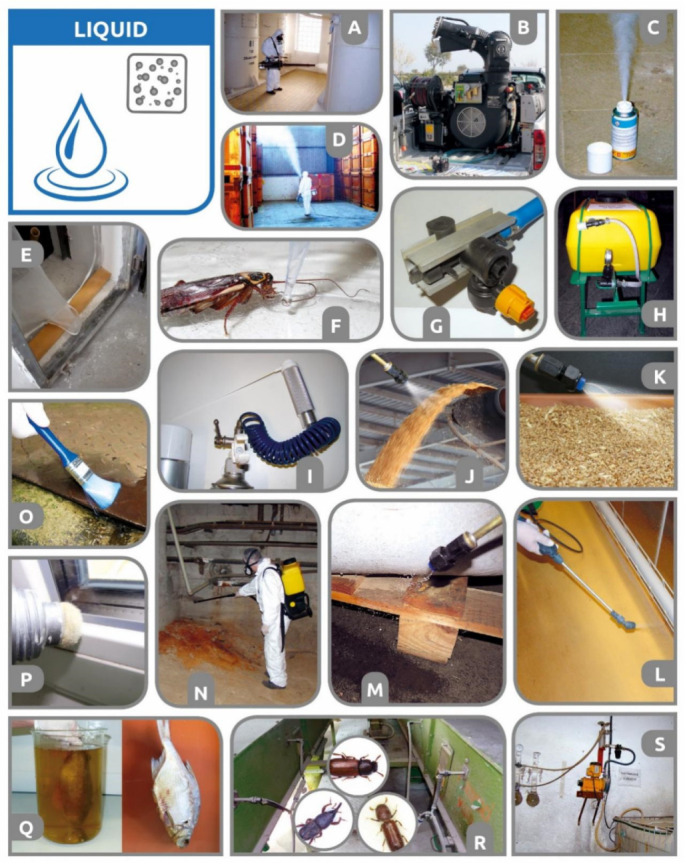
Liquids (**A**) Portable aerosol thermal fogging in food industry silos; (**B**) vehicle-carried cold aerosol applicator; (**C**) cold aerosol container pressurized with propellant; (**D**) cold ULV aerosol application in a store; (**E**) barrier treatment—fluid insecticide-soaked sponge door mat; (**F**) baits—fluid carrier with attractant toxicant or genetic disruptor (dsRNA encapsulated with liposome carriers); (**G**) spray nozzle with device for its attachment to grain conveyor belts; (**H**) mobile compressor and sprayer for grain protectant application; (**I**) dual injection spray/aerosol device; (**J**) visualization of spray protectant applied on grain moving on a conveyer belt; (**K**) visualization of spray/aerosol protectant application on falling grain; (**L**) band barrier or spot spray treatment; (**M**) spray barrier treatment of transport pallets; (**N**) broadcast spray of walls; (**O**) insecticide brushing; (**P**) insecticide sponging; (**Q**) dip application of insecticide protectant on the surface of dried fish; (**R**) multipoint treatment of grain moving on covered conveyer belts (piping is visible; spray nozzles hidden inside covered equipment); (**S**) wall-mounted compressor, insecticide tank, and piping for grain treatment located at the bottom of a grain silo (photographs (**A**–**S**): V. Stejskal; R. Aulicky, T. Vendl).

In practice, for an even distribution of a grain liquid admixture in a commodity, movement of the commodity is required. Conventional treatment consists of continuous spraying of a grain stream with water-diluted concentrates of insecticide emulsions (EC), water-dispersible granules (WG), and suspensions (SC) or encapsulates (CS) [209,250,251]. The treatment is applied to commodities moving on conveyor belts (Figure 3K,R) and inside silos and buckets, screw conveyors, and mobile augers. Protectants may also be applied to streams of falling cereals (Figure 3J). The application is carried out with droplets (perforated dip bars), coarse spraying (special nozzles with holders for fixation on grain transportation technologies—Figure 3G), aerosol misting, or with ultra-low volume (ULV) aerosolization [252]. Smaller sprayer pumps and sprayers (Figure 3H) can be located close to the application sites; more powerful sprayer pumps deliver spray liquid from basements, where large tanks with diluted insecticide are located (Figure 3S). Spray nozzles can be built inside the protective housing (metal covers) of conveyor belts and can be mounted in pairs to enhance spray distribution (Figure 3R). However, the need for available grain conveyors or augers makes the application of liquid protectants as admixture treatments significantly more technologically demanding than the application of dust protectants. Therefore, a special and easier method of liquid protectant treatment (i.e., “top-dress treatment” or “top-dressing”) has been suggested for a static commodity [107,244]. This method of liquid protectant application may not be legal in many countries and for all insecticide products. Arthur [214] warned of the risk that some pests might penetrate through a treated surface in a grain mass of stored grain in a physiological state enabling their oviposition in the untreated layers before they die. In the past, top dressings were mainly used where pyralid moths, such as *Ephestia elutella* (Hübner) and *Plodia interpunctella* (Hübner), have been a problem. Peters [107] stressed that “*top dressing may act as a barrier, preventing insects from entering the grain mass and from feeding on the surface grain. Each time the surface grain is disturbed, such as when probing for moisture or insect samples, the barrier is broken. Retreat disturbed areas with grain protectant*.” In this respect, Athanassiou et al. [253] evaluated the efficacy of spinosad in laboratory bioassays as a surface treatment for wheat to control adult *R. dominica, S. oryzae,* and three psocid species. The results of this laboratory study show that while spinosad has some effectiveness as a layered treatment on a column of wheat, the efficacy will be dependent on the target species, the depth of the treated layer, and the upward or downward mobility of the insect species.

**Dipping of maize cobs.** An alternative to spraying is the dipping of maize cobs [254]. Hodges and Meik [255] showed that maize cobs could be protected against *P. truncatus* infestation if the cut ends of the cobs were dipped in dilute dust or solutions of permethrin (emulsifiable concentrate, wettable powder, or dilute dust).

### 3.2. Dip and Spray Insecticide Coatings for the Protection of Dried or Smoked Fishes and Animal Skins

Dried fishes, meat, and skins should receive effective protection against pests since they are a staple food and a source of protein in many countries. In some rural agricultural settlements and markets, rodent and insect pests (Calliphoridae, Dermestidae, Cleridae—*Necrobia* spp.) are listed among the main causes of damage and spoilage of dry fishes [256] and other types of dried meat and skins [257,258]. Various synthetic or natural active compounds (insecticides/repellents) [259,260,261,262] have been suggested or tested as surface protectants for dried fish and animal skin. Protectants are applied as short-exposure water-based dips (Figure 3Q) [259,263], ULV sprays [264], organic (DDT) or inorganic dust, and synthetic or botanical particles [260] or liquid coatings [265]. However, Islam and Kabir [266] warned that dried fish-related problems are associated not only with pests but also with the harmful chemical protectants applied. For example, Khan and Khan [256] described the risks of residues of DDT that were used as powders directly applied on dried fish in Bangladesh. Golob et al. [259] compared the protection efficacy of water-based dips containing pirimiphos-methyl, iodofenphos, fenitrothion, diflubenzuron, or deltamethrin when protecting dried *Tilapia* spp. fishes against *Dermestes maculatus* Degeer (Dermestidae) infestation. All insecticides provided good protection for two months, but only deltamethrin showed a distinct repellent effect and provided protection for six months. Macquillan and Shipp [258] showed that the organophosphates chlorpyrifos and chlorpyrifos-methyl provided two months of post-treatment protection of sheepskins (>90% mortality) against *D. maculatus*. As an alternative to using synthetic chemicals, the application of diverse natural botanical compounds has been suggested as either a direct dried fish treatment (coatings) and/or a treatment of storage bags. To protect sun-dried fishes against pests, multiple botanical herbal oils, including compounds that are either locally available (e.g., *Detarium microcarpum* seed oil [267]) or generally available (neem [261], garlic, and red chili [262]), have been tested. Don-Pedro [260] discovered that layers of citrus peels and some naturally derived oils (groundnut, traditional coconut, industrial coconut, palm, shark liver oil) have the potential to reduce the risk of dried fish infestation by *D. maculatus* [268]. The absorption of oils by fish surfaces substantially reduces their activity against pest eggs over time. Idris and Funso [269] tested groundnut oil and sodium chloride as protectants of smoked dried fish against infestation of *D. maculatus* and *Necrobia rufipes* (De Geer) (Cleridae) and found that sodium chloride is more effective for long periods of storage. Despite the abundance of published tests on botanical preparations, information about the extent of their current practical usage and field methods of application is largely unavailable in the scientific literature.

### 3.3. Liquid or Aqueous Baits (Traditional Toxic Baits or “Smart Baits” Based on RNA Interference)

Liquid baits are usually composed of water carriers, food attractants (natural/synthetic), and additives. The sugars in these baits facilitate intestinal intake by pests through their sucking, leeching, and feeding behavior. Liquid or semi-liquid baits are available for pests from several taxa, such as ants [270], wasps (e.g., juices from canned chicken with 0.025% fipronil [270,271]), cockroaches (Figure 3F), and *Drosophila* flies [272], which can also be found in warehouses and food factories. Gore and Schal [273] suggested boric acid-sugar solutions as baits for the management of German cockroach (*B. germanica*) infestations. The use and application of these baits are almost identical to those of gel formulations [274], which are already covered in another part of this review (Section 4.1). Therefore, only three emerging bait technologies will be discussed in this section.

The first novelty is the development of liquid baits containing carbohydrates and toxicants targeted at the red flour beetle *T. castaneum* [275]; the authors called this the attractive toxic sugar bait (ATSB) system. The results showed that mannitol supported bait dietary intake and that the active ingredient used (spirotetramat, chlorfenapyr) was lethal when used in the ATSB system. The question of how to deliver these new baits to control storage pests under practical conditions remains to be explored.

The second innovative approach regarding liquid baits is associated with recent discoveries of the insecticide properties of artificial sweeteners (derived from plant extracts or manufactured by chemical synthesis) that may serve as low-energy substitutes for naturally occurring sugars [276]. For example, experiments revealed that a polyalcohol erythritol sweetener was toxic when ingested by some *Drosophila* flies [277] and that it might be an effective insecticide for several genera of ants [278]. Ingestion of sweeteners by insects can lead to significant physiological effects, such as mortality, decreased fecundity, and behavioral changes [276]. For example, it was found that insecticidal polyol sweeteners may induce lethal regurgitation in Diptera pests [279]. The recent review by Lee et al. [276] attempted to summarize evidence that artificial sweeteners could be considered a potentially new pest control approach, useful for the development of a new generation of food baits.

The third novelty was enabled by advances achieved in the field of molecular biology. Recently, a substantially new generation of bait technologies for liquid baits with liposomes as carriers of dsRNA has been suggested. What is the purpose of such a treatment, and how does it affect insects? In recent years, the possibility of using gene disruption technologies (gene silencing)—based on CRISPR and RNA interference—as a method of pest control has been explored [280]. For example, it is expected that RNA interference (RNAi) may overcome pesticide resistance by targeting the expression of genes that contribute to resistance in insects. The routes of dsRNA entry into the insect body include injection, oral applications, and topical/contact spraying applications (“exogenous and endogenous” administration) [281,282]. For the development of baits based on the mechanism of dsRNA gene disruption, the problem is that dsRNA administered orally is not stable in the body of insects. Therefore, a new method was developed [283], which uses liposome vesicles (so-called dsRNA lipoplexes) as carriers of dsRNA molecules. Experiments with cockroaches showed that the protected molecules (lipoplexes), formulated as liquid baits, in the cockroach digestive system successfully triggered lethal RNA interference. Recently, oral delivery-mediated RNAi was first used to silence the LeVgR gene in *Liposcelis entomophila* (Enderlein) (Psocoptera) [284]. The VgR gene may thus become an important potential target to disrupt insect reproduction for pest management through the oral delivery of dsRNA. Owing to these new approaches, there is a real chance that in the future, similar technologies could become the basis for “smart” pest control products delivered via the oral route.

### 3.4. Insecticide Dipping, Impregnation, and Spraying of Bags and Packages

Finished foods, commodities, and seeds can be packed in different shipping containers (e.g., sachets, bags, large bags, and boxes [285]) made of materials including jute, paper, aluminum foil, woven plastic textile, plastic films, etc. However, many types of packaging or materials are not sufficiently resistant to the penetration of mobile stages of pests that infiltrate the packaging through penetration or invasion through small openings and leaks in the packaging [286]. One option for the protection of packaging involves treating their surfaces with insecticides or repellents. In cases where it has been legally feasible, the outer primary or secondary surfaces of the packages (boxes or bags) have been sprayed during storage, or empty bags have been treated by spraying, dipping, and impregnation before storage [287]. However, little information was found on the chemical protective treatment of tertiary packages such as stretch foils and films.

Cotton et al. [288], Hayhurst [289], and Parkin [290] were some of the first researchers to scientifically evaluate the efficacy of chemical (mainly DDT-based) protection of storage bags. The activity of various insecticides on pests was evaluated for various types of packages and packaging materials, including paper bags [288], jute bags [287,291,292,293,294,295,296,297], and bags and packages from synthetic textile or foil materials [287,294,297,298,299,300,301]. Neurotoxic organochlorines [288,290], organophosphates [106], pyrethrins, or pyrethroids have been most frequently suggested as active ingredients for the treatment of bags and other packaging [287,300]. Recently, Papanikolaou et al. [302] suggested the application of thiamethoxam, pirimiphos-methyl, alpha-cypermethrin, and deltamethrin to the surface of storage bag materials (plastic and paper) as an efficient management tool against larvae of *Ephestia kuehniella* Zeller (Pyralidae) and *T. confusum*. There is, however, concern about the migration of neurotoxic insecticides or other chemicals from treated (primary/secondary) packages into commodities or food. Thus, registration appears to be easier in the case of low-toxicity biorational insecticides such as insect growth regulators (IGRs) (methoprene [301]) and botanical preparations.

### 3.5. Liquid Insecticide Aerosols and Mists: Thermal Fogs and Cold Aerosols (“ULV”, “LV”, “HV”)

Multiple definitions of insecticide liquid aerosols can be traced in the literature [36]. Most frequently, aerosols are defined as the physical state of a liquid dispersed—as small droplet particles—in air or another gas or as a suspension of liquid particles in a gas. Himel [303] defined aerosol-type sprays as those having no spray droplets larger than 50 μm. According to the WHO [304] and Sugiura et al. [305], aerosols have a droplet volume median diameter (VMD) of less than 50 µm, whereas mists have a VMD from 50 to 100 µm. Some authors have attempted to distinguish residual sprays from aerosols according to their direct or indirect interactions with arthropod bodies. For example, Hewlett [306] classified three modes of spray delivery of liquid insecticides to insects: “*(1) as a film, when insects come into contact with insecticide previously deposited on surface on which they walk; (2) as mist, the droplets of which impacted on to the insects by slow air currents or by sedimentation, or by the movement of the insects, especially by flight; and (3) as a direct spray, when insecticide is impacted on the insects by droplet movement imparted by the spray gun*”. Depending on the chemical properties of specific insecticide aerosol formulations and the method of application, the active ingredient enters the arthropod body via a combination of inhalation through spiracles and penetration through the integument [307,308,309].

Aerosol-type formulations (active ingredient + additives + carrier) are sold either as ready-to-use products (Figure 3C) or as concentrates to be diluted with water, mineral oil, or diesel oil gas [310]. In contrast to insecticidal gases, aerosols cannot penetrate very narrow cracks, dust layers, or solid or layered materials, which limits the extent of their usage. However, penetration (inside cracks) may be enhanced by the presence of some volatile active compounds (e.g., organophosphate dichlorvos) that may exist both as aerosols and vaporized gas after application [110].

Cold aerosols (Figure 3B–D) and thermal aerosols (thermal fogging—Figure 3A) are the two major technologies for liquid-aerosol insecticide dispersal used throughout the world. Commonly, insecticide aerosol application technologies are also classified according to the volume of applied liquid pesticide and its concentration, as described by Bonds [311]: “*ULV application is the minimum effective volume of the formulated product without any further dilution. If the insecticide is diluted by the operator, the application is considered low volume (LV) or high volume (HV). The insecticide concentration varies depending on the amount of active ingredient in the formulation, ranging from 2% with some of the pyrethroids to 95% with the organophosphates*.” The “ULV” type of aerosol treatment is created mostly by cold fogging devices. “LV” and “HV” aerosol applications are typical for thermal fogging devices and only to a limited extent for the specific use of cold-aerosol devices [311]. Depending on the water content, aerosols may occasionally also be classified as “dry” or “wet” fogs [312,313].

**Figure 4:**
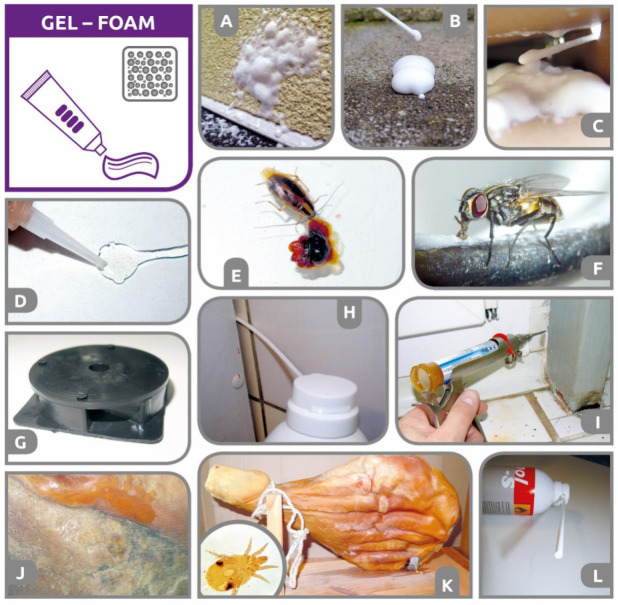
Gels and foams. (**A**,**B**) Insecticide foams can be applied on vertical and horizontal surfaces; (**C**) foam injected into cracks and crevices and voids; (**D**–**F**) gel and/or foam bait for ants, cockroaches, and flies, respectively; (**G**) bait administered in a plastic resistant box; (**H**) foams and gels that can be applied from ready-to-use pressurized containers; (**I**) gel bait application into cracks and crevices using injection guns; (**J**) detail of protective gels applied on ham directly or on ham nets; (**K**) ham surface gel protection against mites; (**L**) detail of a plastic tube gel or foam injector (photographs (**A**–**L**): V. Stejskal; R. Aulicky, T. Vendl).

**History and current status of insecticide aerosols**. For almost 100 years, aerosolized insecticides have been used against nuisance and public health insects and household or storage pests. The historical development was technically divergent for spray-application devices, generating either cold or thermal aerosols. Regarding the earliest devices for the application of cold (natural pyrethrin-based) aerosols, Matthews [314] listed either twin fluid compressed air “paint-sprayers”, or “piston-gun”-type hand-held devices. The latter were known as “Flit-guns”, “Fly-Tox-guns” (Figure 4A), and similar products. Such devices were able to atomize fluid to some extent into fine spray droplets that remained airborne long enough to kill insects active in the treated space [314]. They were therefore considered predecessors of the modern types of small handheld pressurized containers for non-professional/professional use (insecticide bombs, total release foggers, etc.) or of large cylinders for industrial use [35,110]. Containers or cylinders are pre-pressurized/pressurizable in combination with various gases or pressured air as propellants, and they may eventually be incorporated in automated aerosol application systems [110]. Automatic systems are constituted either from a set of separate application units [315] or by a centrally placed container that is connected with a network of tubing, ending with nozzles to which insecticide fluid is delivered under pressure (e.g., CO_2_ propellant) [316]. The new generations of various motorized (hand-held, transportable, vehicle-mounted) devices for cold aerosol/mist applications for public health and urban pests originated from modified agricultural equipment developed in the 1950s [317]. It is claimed that the concept of insecticide thermal fogging originated from military technologies in the early 1940s [87,318] For example, Matthews [314] stated that thermal pesticide devices either evolved from military smoke-screening generators (i.e., devices based on heat exchangers) or were inspired by research associated with the German rocket V1 (i.e., devices based on exhaust pulse jet engines). Collins and Glasgow [150] described two early historical types of generators that were both used for thermal fogging and designed to disperse emulsions or suspensions. The first device, known as the “Hochberg-LaMer thermal aerosol generator”, was based upon modifications of earlier military fog-oil screening “smoke” generators. It was considered a “wet” aerosol fog generator, since it used water + oil insecticide liquid. Superheated steam, under controlled temperature and pressure, broke up the oil solution into droplets of the desired particle size. The second early historical thermal device was described [150] as the Todd Thermal Aerosol Insecticide Fog Generator (“Todd Insecticide Fog Applicator”—TIFA), which originated from a modified oil-fog generator developed and manufactured largely for use by the Navy. This device produced the “dry” type of aerosols since no water was added. An oil solution of the insecticide entered a mixing chamber, where the droplets were heated in a blast of hot air maintained at a controllable temperature and pressure and thus dispersed into smaller droplets when discharged at atmospheric pressure. Recent and independent comparisons of various thermal or cold aerosol devices and systems—in terms of their performance (e.g., application rate per volume and time unit) and properties of the generated insecticide aerosol (size of droplets and their spectra, spatial distribution, active ingredient thermal degradation, etc.)—are available in various peer-reviewed publications [36,319,320,321] or in WHO resources [304].

One of the first industrial-scale uses of aerosols to control stored-product pests (namely, *P. interpunctella* and *E. elutella*) was described [322] by Charles Potter, an influential entomologist from Imperial College, London [323]. He proposed not only a new prototype of original fogging machinery but also a new method of frequently replicated applications of atomized white oil-pyrethrum fluid. Due to poor access to pyrethrum (especially in Europe during the Second World War), thiocyanate was formulated (Thanite^®^) as one of the first organic synthetic compounds commercially used as aerosols [324]. In parallel, aerosol formulations based on chlorinated pesticides were developed. In the USA, DDT thermal fog mixtures (DDT, DDT + pyrethrins, DDT + tetrachloride) were successfully tested to control clothing moths in wool storage warehouses in 1945 [150]. It was found that DDT aerosols not only had 100% direct contact efficacy on adult moths but also certain residual efficacy, manifested in killing some of the subsequently emerging adults and larvae. Extensive research, mainly on aerosol formulations of dichlorvos (DDVP) and synergistic pyrethrins, was conducted in the 1960s–1980s to control storage and food industry pests [313,325]. Later, Bell [326] called the aerosol method somewhat outdated and considered that its increasing popularity in the food industry was due to a misleading association with gas fumigation. He even claimed that the insecticide aerosol “*does not deal with the root problem of infestation and is best regarded as a cosmetic action*”. In contrast to this opinion, continually emerging research and practical interest in insecticide aerosols can be seen from the late 1990s until today [327,328]. This renewed interest was initially triggered by a ban on methyl bromide in mills and later was boosted by the discovery of the field residual action of some aerosol mixtures [329,330]. As a result of recent extensive research activities, a number of published original works are now available concerning research into the effectiveness (laboratory and field) of various types of aerosols and their methods of application [321]. These works include studies on different species and developmental stages of storage pests regarding various environmental factors (temperature, structure, obstructed/unobstructed exposure, presence of food material on increased survival, etc.). Their overview can be found in several specialized reviews [34,110,331,332]. It should be noted at this point that David [312,333] and David and Bracey [334] were among the first scientists who systematically analyzed factors (e.g., size of droplets) influencing the interactions of insecticidal mists with flying insects.

Regarding stored-product pests, it is clear from the available literature that research into the effectiveness of aerosols has been carried out mainly on beetles [329,330,332], psocids [335,336], and moths [337,338,339], whereas data for mites are missing. Little published research, especially field testing, is available on food-industry pests of hygienic importance, such as cockroaches, flies, and wasps [48,305,340,341]. Although some reports indicated considerable activity of some aerosols on cockroaches under the tested conditions [48,305,341,342], others reported complete ineffectiveness (total release foggers [343]) or at least inadequate control (ULV generators [340]) without supplemental applications of other insecticide formulations, such as baits or residual sprays. However, cockroach populations (*Periplaneta* spp.—Blattodea) hiding in sewers may not be accessible to bait or spray treatments. Therefore, Chadwick and Shaw [342] tested the efficacy of pyrethroid aerosol insecticide (bioresmethrin + PBO in kerosene) applied as thermal fogging into sewer underground systems. They found that an acceptable degree of control might be obtained if thorough and replicated treatments were used. Moreover, promising control results were also reported for the combination of aerosol-based flushing (pyrethrins + PBO) of cockroaches and their concurrent physical removal using a vacuum cleaner [344,345]. For example, Kaakeh and Bennett [344] achieved an 80% reduction in the consequent German cockroach trap catch after the “flushing-and-vacuuming” treatment of the infested apartments.

**Aerosols: Active ingredients, application formulations and systems.** Active ingredients labelled for aerosol formulations mainly comprise pyrethrins and pyrethroids (D-allethrin, tetramethrin, vaporthrin, deltamethrin, cypermethrin, etc.), organophosphates (DDVP—dichlorvos, fenitrothion, etc.), and to a lesser extent carbamates. The slow-acting insect growth regulators (IGRs/IGDs) methoprene, hydroprene, and pyriproxyfen are frequently formulated either alone or as a mixture with conventional rapid-acting neurotoxic compounds [110,332]. Few pyrrole or neonicotinoid insecticide compounds have been registered for space applications. Similarly, we were not able to find any published documentation available on commercially used botanical insecticides/acaricides as aerosols at the industrial scale.

Most active ingredients are formulated as oil-based preparations (O), ready-to-use emulsion concentrates (EC), water-based concentrates, or ready-to-use ULV formulations (EW) [310,346]. Micro-encapsulated formulations (SC/ME) of fenitrothion, chlorpyrifos, and diazinon were also found to be applicable as aerosols [347]. According to Einam [348], the droplets must stay viable (chemically protected) in the air long enough to hit the insect targets. However, some “true” water-based formulations (e.g., pyrethrum or deltamethrin) do not require chemical protection. Remedial insecticide aerosol treatments are frequently required by water-based aerosols (pyrethrin + PBO -AquaPy™) to control *E. kuehniella* in some European mills [346].

Several authors have shown that the droplet size of aerosols is of critical importance regarding their insecticidal efficacy [110,312,332,336]. Therefore, Asuncion et al. [321] evaluated the characteristics (droplet size, dispersion and deposition of aerosols) of six aerosol delivery systems and devices in a simulated stored-product facility. They included two handheld sprayers and compressed gas sprayer systems fitted with two types of manifolds and two types of nozzles. The authors found that the spray systems differed significantly in spray characteristics. The compressed gas sprayers generated significantly smaller droplets, more uniform droplet size distributions, and better spray coverage than the handheld sprayers. The ellipsoidal nozzle produced significantly smaller droplets than the circular nozzle.

### 3.6. Surface Spray, Brush or Sponge Applications, Leaving Residual Insecticide Deposits

The objective of the residual type of treatment is to establish effective insecticide residual deposits on the treated surfaces [34,38,332]. The deposits are formed from single droplets [349], droplet/capsule aggregations [350], or films [351,352]. The deposits are usually achieved through spray (Figure 3L), brush (Figure 3O), or sponge application (Figure 3P) of an insecticide liquid. Brushing is typically used for the application of lacquer formulations [324], whereas coarse spray is typically used for the delivery of emulsions and suspensions. Sprays are defined as the physical state of a liquid dispersed as relatively large (most frequently ranging from 100 to 600 μm) droplet particles in air [36]. The thickness of the liquid insecticide continuous film on the treated non-absorbing surface is approximately 50 μm [324]. Control is achieved when a lethal amount of deposits is picked up on the appendices (adult tarsal or antennal structures, larval pseudopods) or insect/mite body surface of the passing/resting arthropods [349]. Sufficient amounts of deposited insecticide residues must be delivered to the targeted surfaces of walls, floors, or building structural components to ensure control of all mobile pest stages encountering the treated surface for the subsequent days or weeks following application [38].

**Evolution of the concept of the industrial residual pesticide spray-formulation.** From ancient times until the 20th century, agricultural pesticides were mainly prepared by farmers for themselves. To improve the field performance of such home-made insecticidal sprays, chemical engineers have proposed the addition of other chemicals, known as stickers, wetting agents, and spreaders [26]. Munro [26] pointed out that each manufacturer had a specific and “secret” formulation for which proprietary rights were claimed; such types of proprietary rights were known from Victorian times as “Keatings”. New demands on technological quality and standardization of the process of pesticide production and usage have gradually evolved into “manufactured” formulations of insecticides. The initial stage of evolution of a scientific concept of residual-surface-deposition of modern liquid insecticides in storage and urban environments was described and mainly credited to C. Potter [323] (from Imperial College, London) by Pradhan [352] as follows: “The importance of the residual effects of insecticidal sprays and dusts has been realised in varying degrees by most of the serious workers in the field of insecticides and fungicides [351,353] but he (C. Potter) appears to have been the first (1938) to give primary importance to it. He proved that it was desirable to spray warehouses not necessarily with the object of hitting the insect directly during treatment but mainly to deposit a protective film on exposed surfaces, so that the moths emerging or flying out of crevices subsequent to spraying might continue to get fatal doses of insecticide on settling on that film. This conception has been definitely consolidated [306,354,355,356,357] into what is described as the ‘film technique’, as distinct from ‘direct spraying’, for the biological evaluation of insecticide toxicity”.

#### 3.6.1. Formulations and Active Compounds Used as Surface-Residual Sprays

**Early history of residual sprays**. In 1922, Swenk [358] recommended very simple and limited chemical measures for a surface structural treatment of a commodity store in the USA (Nebraska) before newly harvested grain was added: “… *the floors, walls, and ceilings of the bins should be thoroughly cleaned, and the floors sprinkled with air-slacked lime (which should be again removed before the grain is stored) or sprayed with benzine or gasoline (care being taken to keep away all fire and lights until the liquid has evaporated and the vapors have disappeared); or, if necessary, the whole granary fumigated with carbon bisulfide or sulfur dioxide fumes*.” The German scientist Friedrich Zacher (from the Institut für Vorrats- und Pflanzenschutz, Berlin-Dahlem, Germany) summarized [359] the substances historically used as insecticides for surface spraying of the structures of empty warehouses in the late 1920s. These substances included tar products, lime milk, aniline oil, paraffin, a mixture of hexachloroethane + paraffin, sulfur liquid, tobacco extracts, soap brew, soap brew with an extract from Quassia wood (*Picrasma excelsa*), etc. It is noteworthy that the professional literature of that time presented application formulations in the form of instructions or recipes, such as 2 kg soap + 1 L tobacco extract + 100 L water [359]. In the USA, Robinson [360] published an extensive overview of agricultural spray insecticides and their mixtures, as well as instructions for the preparation of “home-made insecticide solutions” in 1935. The listed sprays were based mainly on inorganic compounds, organic or inorganic oils, and botanical extracts. However, he stressed that arsenate of lead and other arsenicals cannot be home prepared. Robinson [360] also provided one of the first compatibility charts, indicating which commonly used insecticides may be mixed safely in a single spray tank. Later, in 1947, Shepard [211] recommended the following guidelines for spraying grain-store floors and walls: “*Cracks should be treated by squirting oil or kerosene into them from an oil can. Kerosene or turpentine may be used as sprays. Many of these sprays have a strong odour that may to a certain extent repel insects...but grain absorbs odours*”. In 1945, McDaniel [361] provided an overview of recipe-type instructions for the preparation of home-made insecticide mixtures that were used for the control of pest flies or cockroaches in diary food-industry facilities; e.g., “*mix 1 part formaline with 19 parts of water*”, or “*mix pyrethrum with sodium fluoride and pyrophilite in a ratio 20%:10%:70%, where 2% thiocyanate may be substituted for pyrethrum*”. Although pyrethrum is odorless, Munro [26] complained that instability limits its uses since it was observed to readily decompose when exposed to air, sunshine, and hot environmental conditions. Rotenone was also found to be unstable when applied as a residual insecticide deposit [362]. It cannot be overlooked that the current problems (e.g., “recipe-like formulations or formulas”, aromatic smell, instability) with the preparation of home-made natural insecticides eerily echo approaches, problems, and events that took place nearly 100 years ago in early periods of the chemical control of stored-product and food industry pests [26,211,359,363].

**Transition from old to modern compounds and formulations**. As indicated above, the beginnings of the modern residual chemical protection of warehouses and food operations are associated with oil/water-based formulations of natural pyrethrum, rotenone, and nicotine. In 1935, because of the instability of botanical extracts, the first patent protecting the combination of natural and synthetic (thiocyano) organic insecticides in the USA was applied for [364]. Due to the absence of pyrethrum during World War II, synthetic insecticide formulations purely based on organic thiocyanates (e.g., Lethane 384^®^, Lethane A70^®^) were developed and marketed. Due to their unpleasant odor and skin irritation, these products were gradually replaced by chlorinated hydrocarbons, including DDT, gamma HCH, etc. [26,324]. Chlorinated hydrocarbons gradually became generally available and adopted during and after World War II [365]. The post-war generations of active ingredients further included carbamate (propoxur, bendiocarb, etc.), organophosphate (DDVP-dichlorvos, fenitrothion, acephate, diazinon, chlorpyrifos—methyl/ethyl, pirimiphos-methyl, etc.), and pyrethroids (D-allethrin, tetramethrin, vaporthrin, deltamethrin, cypermethrin, fenvalerate, l-cyhalothrin, resmethrin, etc.) groups [212]. Later, the slowly acting insect growth regulators/disruptors (IGDs/IGRs) pyriproxyfen, r/s-methoprene, s-methoprene, cyromazine, and hydroprene were introduced as residual surface sprays [35,366]. Tank-mixing of juvenile hormone analogues (JHA) and neurotoxic (e.g., pyrethroids) spray insecticides has been used since 1980 for cockroach control in the USA [366]. The most recent groups are represented by compounds such as pyrazole and pyrrole pesticides (e.g., fipronil, chlorfenapyr) or neonicotinoids (e.g., thiamethoxam, acetamiprid) [367,368,369]. However, in food industry facilities, pyrroles or neonicotinoids are mainly used as cockroach/ant baits due to their long-term persistence in the environment [370]. For stored-product pests, the most chemically diverse contact insecticide group is botanical essential oils, which mainly originate from four plant families: Lamiaceae, Asteraceae, Rutaceae, and Myrtaceae [69]. Campolo [69] stressed that the unambiguous classification of an individual botanical insecticide as a contact insecticide is difficult since essential oils frequently exhibit multiple routes of insect body entry.

Spray insecticide liquids are commercially available as either ready-to-use or concentrated formulations; the latter are diluted on-site, mostly by water. Several types of concentrates of synthetic pesticides are used to control agricultural and urban pests [212,331,371,372,373,374]; they commonly include emulsified concentrates (EC), nanoemulsions (oil in water—O/W; water in oil—W/O; and bi-continuous nanoemulsions [375]), wettable powders (WP), suspension concentrates (SC), flowable concentrates (FC), and micro-encapsulated concentrates (ME; SC) [376,377,378]. Stejskal et al. [377] found significant differences in bioavailability (for *B. germanica*) on porous and the non-porous surfaces in various micro-encapsulated preparations depending on the size of the microcapsules. Comparison of their microcapsule size spectra revealed that formulations containing larger microcapsules had higher efficacy on porous surfaces than formulations with smaller microcapsules. Nanoemulsions are colloidal systems—also known as miniemulsions, sub-micron emulsions, or ultrafine emulsions—in which the emulsified particle size is between 20 and 500 nm [375]. Lacquers (LACs), as ready-to-use formulations, were established as special insecticide preparations for urban environments to control crawling pests such as cockroaches [379,380]. Busvine [91] described technical evolution and properties of insecticide lacquers as follows: “*In the years immediately following the Second World War, the newly introduced synthetic insecticides were tried out in diverse formulations. One idea was to mix DDT with whitewash, distemper or paint to obtain insecticidal wall decorations. Unfortunately, it was found that much of the contact action was lost by the DDT being masked by whitewash particles or embedded in the paint film. Then it was discovered that, if a sufficiently high concentration of DDT was included in certain oil-bound paints, the insecticide would migrate to the surface and become extruded as a bloom of crystal. Furthermore, this bloom would be renewed, if wiped away. Subsequently, it was found that even better results could be obtained with insecticides incorporated in certain synthetic resins, which produced insecticidal lacquers…The lacquer may be applied to any clean, non-porous surface (hard paint, glazed tiles, glass). Paintwork less than three months old should not be treated, however, and metals should be pre-treated with a primer to avoid corrosion. It is also inadvisable to treat surfaces constantly wet or frequently washed*.” On the contrary, Cornwell [324] stressed that lacquers are optimized for situations where sprays or dusts are lost or rendered useless by frequent washing and cleaning, and the condition for usage was that the treated object had to be ventilated due to solvent evaporation and odor. Reid [379] found that a laboratory surface treated (painted) with urea-formaldehyde resins (containing DDT, HCH, aldrin, and dieldrin) showed residual insecticide activity against the speckled cockroach (*Nauphoeta cinerea* (Olivier) (Blattodea)) and dark flour beetle (*Tribolium destructor* Uyttenboogaart (Tenebrionidae)) for more than 11 months. 

**Spray formulations of natural compounds**. Natural insecticides are frequently formulated as oil formulations (O), emulsions (EC), or nanoemulsions. Campolo [69] reported that the major concern regarding the practical usage of many botanical essential oils is associated with their low persistence after application (e.g., rotenone [362] or pyrethrum [364]). To overcome this disadvantage, there are efforts to develop polymer-based micro- or nano-encapsulated release formulations [381,382]. However, the use of encapsulated formulations—with a 9:1 ratio of active ingredient to polymer content [383]—should not counteract the mitigation of micro-plastic environmental contamination. Nevertheless, newly developed biodegradable microcapsules [378] seem to be promising and environmentally friendly solutions to this problem.

**Repellents as structural sprays (barrier, push-and-pull, and repellent + cleaner treatments).** The early history of insect repellents was briefly described by Peterson and Coats [384]. They stated that “…*the use of insect repellent compounds dates back to antiquity, when various plant oils, smokes, tars,* etc. *were used to displace or kill insects. Before the Second World War, there were only four principal repellents: oil of citronella, sometimes used as a hair dressing for head lice, dimethyl phthalate, discovered in 1929, Indalone^®^, which was patented in 1937 and Rutgers 612, which became available in 1939…. In 1953, the insect repellent properties of N,N-diethyl-m-toluamide (DEET) were discovered and the first DEET product was introduced in 1956*”. Residual repellents may be applied on surfaces as general, spot or crack, and crevice treatments in order to repel pests from undesired areas, shelters, and harborages. Repellents may also be considered as barrier treatments of potential entry points into buildings [385]. A special approach, combining the application of repellents and attractants, is called the “push-and-pull method”. Repellents push the pests from unwanted areas, whereas attractants pull them into the areas treated by insecticide sprays or baits. Push-and-pull strategies were originally developed for agricultural pests [386] and later suggested for the control of cockroaches [387] and flies [388]. Another concept is a combination of repellents with cleaning chemicals. For example, in Europe cockroach repellent floor cleaner (Ajax Expel^®^—Colgate-Palmolive (New York, USA)) was released, the active ingredient of which was N-methyl neodecanamide [384]. Brenner et al. [389] concluded that cockroaches were less likely to re-infest previously occupied areas after treatment with the repellent cleaner. As a non-synthetic alternative, natural essential oils (EOs) were tested (surface or topical applications) against cockroaches and stored products beetles [384,390,391,392]. Karr and Coats [393] demonstrated that fragments of Osage orange fruit and its hexane and methanol extracts were repellent to the *Blattella germanica* (Linnaeus, 1767). The obtained results for one pest species cannot be always be generalized for related pest species since different stages and species vary in their sensitivity to EO repellents. Milled red cedar flake boards were found to be repellent to *B. germanica*, but not to *Periplaneta americana* (Linnaeus, 1758) or *Supella longipalpa* (Fabricius, 1798) [394]. Since many EOs may be volatile and not very persistent after their application, the development of stable and slow-release EO repellent formulations (e.g., encapsulated) are currently needed for their widespread practical industrial implementation.

#### 3.6.2. Biotic and Abiotic Factors Influencing the Efficacy of Insecticide Deposits

A vast number of studies on the efficacy of synthetic residual insecticide deposits [35,38,332] have been published on Blattodea [38,395], Hymenoptera [396], Coleoptera [34,367,397,398,399,400,401], Psocoptera [402,403], and Acarina [404,405,406,407]. Relatively less abundant are studies on Diptera [408], Lepidoptera [401,409], Zygentoma, and Orthoptera [410]. Most data on botanical insecticides are available on Coleoptera, followed by Lepidoptera, Psocoptera, Acari [69], and Blattodea [38,395]. The efficacy of both synthetic and natural insecticide deposits is influenced by multiple biotic and abiotic factors [401]. The most studied environmental factors include the active ingredient, formulation [411], age of deposits [412], temperature, humidity [401], type of treated surface [377], and surface contamination and dirtiness. Biotic factors include the species, developmental stage, and population sensitivity. Moreover, biotic factors may also include the factor of human pesticide applicators. Stejskal and Aulicky [413] quantified how variations in individual human behavior can affect the application time and dosage (i.e., over-dosage or under-dosage) of residual insecticides to control storage and food industry pests. Stejskal [414] demonstrated that insecticide application may even be described by a functional/aggregative (“predator-prey”) response of applicators to various pest densities. For the targeted application of residual insecticides, accurate species identification is required, since closely related stored-product pests may show differential susceptibilities to insecticides (e.g., *Liposcelis* spp. [402], *Tribolium* spp. [415]). Laboratory studies on ants and cockroaches are frequently followed by field validations [38,416,417]. The effects of aerosols and fumigants on stored-product insect pests under field conditions of store flourmills are well documented [54,201,418,419,420]. In contrast, with rare exceptions [421,422], most studies concerning the activity of residual insecticides on stored-product arthropods are mostly performed under laboratory conditions [35,332,401]. However, laboratory studies (especially forced-contact and no-choice tests) may not be able to reflect or simulate natural field conditions because of the possible involvement of physiological or behavioral components, including resistance, frequency of dispersal, movement patterns, avoidance behavior due to insecticide repellence, etc. [25,423,424,425]. Among the “field exceptions” are the two pilot studies conducted in the USA [421,422] that used two application strategies for residual treatment of the artificially infested experimental warehouses with *T. castaneum* by (S)-hydroprene and cyfluthrin. The insecticides were applied either around the inside perimeter of the warehouse or in a band around the base of shelf units containing the infested flour patches. The evaluation of contact efficacy was based on insect captures in food- and pheromone-baited pitfall traps. The number of adults captured in pitfall traps reflected adult mortality in cyfluthrin-treated warehouses. Although there were significantly more dead adults in warehouses treated with cyfluthrin than with (s)-hydroprene or water control treatment, the food patch samples showed no detectable differences in the quantity of larvae, pupae, or adults among any treatments.

The lack of convincing field data regarding sufficient insecticide efficacy even led Gudrups et al. [426] to skepticism about the cost efficacy of using surface spraying with neurotoxic insecticides to control stored-product pests in grain stores located in tropical and subtropical regions. The authors expressed concern that continuous exposure of populations to sub-lethal insecticide doses might lead to an increase in resistance, and they suggested, as a solution to this problem, applications of non-residual insecticides integrated with commodity fumigations [426].

#### 3.6.3. Equipment and Types of Spray or Brush Applications (Broadcast, Spot, Crack-And-Crevice, Barrier, Direct, and Special Treatments)

According to Matthews [427], the earliest models of industrially used liquid application devices had two hoses attached to the tank-bottom part of a “knapsack sprayer” construction. The spray liquid was pressed by gravity from the tank bottom to the tip of each hose, equipped with a “sprinkler nozzle” through which pesticide was sprayed on the treated surfaces. The first predecessor of modern knapsack sprayers, based on the pump compression principle, was designed in France in the 1880s [427]. Currently, deposits of residual insecticides for the control of urban and storage pests are most commonly delivered by a broad variety of hand-held, portable, or vehicle-mounted sprayers equipped with flat-fan or pin-stream nozzles or injectors [28]. Sprayers are pressurized manually or by electric (e.g., energy supplied via a cable or an attached battery) or ignition engine pumps. General instructions for the manipulation and use of application equipment are provided by their manufacturers. Additional information can be found in specialized pest control books and textbooks (e.g., [26,27,28,36,37,428]) or in various WHO and FAO publications and manuals. A comprehensive overview and practical guide for insecticide application equipment to control urban pests was prepared by Robinson [28]. In contrast to insecticide aerosol generators [321], no specific performance comparative study has been published on devices for the application of residual sprays used for the control of stored-product pests.

In most countries worldwide, the permitted methods of using specific products (resulting from the process of their legal registration) are provided on the label for the practical user. Generally, the most commonly used types of spray applications include (i) general broadcast treatment of surfaces (Figure 3N) [401], (ii) spot spray treatment (a single “spot application” usually does not exceed the area of two square feet) and crack-and-crevice spray treatment (injection, drill-and-treat) (Figure 3I) [401,429,430,431,432,433,434,435], (iii) barrier and indoor/outdoor perimeter surface spray (Figure 3L), brush (Figure 3O) or sponge (Figure 3P) insecticide treatment [324] (depending on legislation, insecticide may also be applied as a barrier spray around or on transport pallets—Figure 3M), (iv) direct contact treatment of pest body surfaces by coarse sprays [28,38,436], and (v) window glass sprays. In the past, Dove [437] recommended the application of residual sprays on the surface of glass windows, where flies and other insects are attracted by outside daylight.

Currently, coarse direct-spray treatments of pests are used less commonly than indirect methods in practice [26]. Some botanical oils [69], petrol oils [436], and silicone (organosilicone) polymer surfactants [438,439,440] have been tested with promising results as direct sprays [306] on urban or storage arthropods. Various fatty acid salts, soaps, and surfactants showed insecticide activity against German cockroaches in laboratory settings [441,442]. However, fatty acid salt liquids (1%–2%) were effective in killing *B. germanica* and *P. americana* cockroaches only when the insects were thoroughly wetted [441]. Direct treatment of arthropods with a low dose of an oil results in cuticle dewaxation and oil penetration through the cuticle into the insect body, where it directly lethally affects cells, whereas a high-dose oil treatment may also involve a suffocation effect through the blockage of spiracles and the tracheal system [19,307].

Indirect coarse spray insecticide applications are the most frequently used in commodity stores and food industry facilities as general, spot, and crack-and-crevice treatments. For the food industry, the USA has even legislatively defined some indirect methods of applying residual products, such as general, spot, and crack-and-crevice treatments [401,443]. Zettler and Arthur [444] emphasized that it may be difficult to control infestations inside mills, food and feed industry facilities, and storage facilities because the vast proportion of insects is hidden in refuges [445,446] and the duration of exposure/contact with openly deposited insecticide residues may be limited due to pest movement and avoidance behavior [25,447,448]. Therefore, instead of broadcasting residual surface treatments over a large area, insecticides are specifically targeted to selected sites within a facility. Targeted spray barrier treatment may be considered to prevent pest immigration among buildings or pest migration among pallets with infested and non-infested commodities. Along with mechanical sanitation [449], spot insecticide spray treatment may be employed to control aggregations [450] of pests in food industry premises and empty grain stores. According to Arthur [332], empty grain stores and bins can be considered a structure; thus, residual insecticides are often used for the general broadcast treatment of flooring before the loading of new grain, known as “pre-binning treatment”. The pesticides used for structural treatment have different registration and labels from those used for direct grain admixture treatment; therefore, they must not come into contact with stored commodities or food/feed. Spray treatment of empty stores and bins is advised to be performed at least two weeks prior to adding new commodities (https://www.sites.ext.vt.edu/newsletter-archive/cses/2005-10/grain.html (accessed on 24 June 2021)).

**Special residual insecticide treatments (mat barriers, hanging cords, strips, and hanging-droplets)**. Several other special methods of delivery of liquid residual insecticide have been historically developed that do not fit the previously described traditional classifications. For example, in China, a concept of insecticide-treated porous door mats (made from sponge-like materials and soaked with insecticides) that prolong insecticide residual action was developed (Figure 3P). These insecticide doormats serve as insecticide barriers to prevent walking insects from entering grain stores outdoors. To control flying insects, residual insecticides may be applied not only on floor and wall surfaces but also on horizontally attached cords or on vertically hung strips, boards, or window mesh [451]. A special case of insecticide contact deposits for Diptera control was described by Gostick et al. [452] as “hanging droplets”. The principle is that relatively large oil-based drops hang on a thin vertical wire, and contact of a fly with the liquid drop is associated with the transfer of a substantial volume of liquid onto the insect’s body.

## 4. Insecticide Gel and Foam Application Formulations

### 4.1. Gel and Paste Baits

Currently, insecticide gels and pastes belong among the most commonly used bait formulations. Insecticide gels have a generally higher water content (39–80%) than that of pastes (14–30%) [453]. Baits belong to the category of “passive preparations”, which means that they require pest activity to find them and consume them (Figure 4E,F). Therefore, in addition to toxic substances, baits must contain compounds (attractants/phagostimulants) that highly enhance their attractiveness and palatability. Bait activity can also be associated with the soft physical structure and/or high content of water in the bait. The criteria regarding the required bait properties are usually fulfilled by most traditional or chemical (frequently hydrophilic) gels and pastes, micro-encapsulated oil baits [454], and polyacrylate hydrogels (ants [455], wasps [456]). The attractiveness can be enhanced by pheromone addition into baits [457], which is in practice known as the “pheromone-assisted baiting technique” [458]. Ready-to-use forms of gel and paste baits (Figure 4D–F) are delivered to the destination using pressurized propellant-based containers with plastic tube injectors (Figure 4H) or pressure or vacuum injection gun-type applicators (Figure 4I) with removable injection tips. Another form is the administration of baits in tamper-resistant box stations that are ready to use or are filled into empty boxes on-site (Figure 4D). Plastic box stations provide some degree of protection against environmental contamination and consumption by non-target organisms [38]. Miller and Smith [459] developed methods of bait application into wax paper called “bait tacos“; bait drops were inserted—using a bait-gun—into square wax paper pieces folded to form a semi-open triangles. “Prey-baiting” or “Trojan horse approach” is considered an innovative bait pesticide delivery method for the control of ants (*Pachycondyla chinensis* and *Linepithema humile*) [460,461].

Baits within the IPM framework have the potential to greatly reduce the amount of spray insecticides needed for pest control [462] and to reduce the accumulated amount of residues remaining in the indoor environment [463]. Compared to residual spray formulations, baits are relatively less toxic, odorless, and may be applied in minute amounts to areas where residual spray is not permissible [453]. However, in the sensitive environment of food operations, some hygiene auditing systems require the disposal of baits after use.

There are almost no published records on the use of baits to control Coleoptera, Lepidoptera, Psocoptera, and Acari in storage. The only exceptions include laboratory reports on *Tribolium* sp. baits [275] and the evaluation of various commercial baits on firebrats and silverfishes (Zygentoma) [464,465,466,467] and psocids [468,469].

Historically, “home-made types” of toxic bait paste were most commonly prepared [324,453]. The efficacy of these homemade formulations was highly variable because they were prepared by individual pest control operators in small batches from locally available food components [38]. Tee and Lee [453] stated that “*the first cockroach bait was available commercially in 1896, when phosphorous was added to a sweetened flour paste and marketed to kill cockroaches in the USA and UK. Prior to that, do-it-yourself cockroach bait was made by mixing 1 part plaster of Paris with 3–4 parts flour, and this mixture functioned as a stomach poison*.” To control silverfishes/fishmoths, pastes containing barium fluorosilicate, sugar, flour, Arabic gum, and water were painted onto wooden surfaces [470]. Alternatively, phosphorus paste was mixed with bread, fruits, and rotting vegetables and then applied as a home-made bait (on paper or wooden boards) to control cockroaches [361,471]. Later, boric acid and neurotic insecticides such as carbamates (e.g., propoxur, methomyl) and organophosphates (e.g., chlordecone, chlorpyrifos) were used for the control of various insect species such as flies, ants, and cockroaches [453,472]. In the last several decades, new generations of baits have been established. These mainly contained relatively slow-acting compounds that included some neurotoxic insecticides (phenylpyrrazoles—e.g., fipronil; neonicotioids—e.g., imidacloprid, dinotefuran; oxadiazines—indoxacarb [453,473]); cell-respiration and energy production disruptors (amidinohydrazones—e.g., hydramethylnon [474]); uncouplers of oxidative phosphorylation, (fluorinated sulfonamides e.g., sulfluramid) [453,475]; disruptors of electrical activity in nerve and muscle cells (avermectins—e.g., emamectine benzoate, abamectine [476,477]); anthranilic diamide ryanodine receptor activators (e.g., cyantraniliprole [472,478]); and insect growth regulators/disruptors (IGRs/IGDs) (analogues of juvenile hormone (juvenile hormone agonists (JHA)), chitin synthesis inhibitors (CSIs), or ecdysone inhibitors (ecdysteroide antagonists) (EIs/EAs) [233,477,478,479,480]). More recently, cockroach baits based on locally available natural compounds have been explored [481].

The most extensive current application of the modern generation of baits mainly concerns food and urban pests, such as cockroaches (Blattodea), flies (Diptera) [472], and ants and wasps (Hymenoptera) [456]. For some of these pests, it has been reported that the accelerated activity of baits is based on toxicant horizontal/secondary transmission. Secondary or even tertiary [482] intoxication occurs when the deposited feces [483], vomitus substances, or carcasses [484,485,486,487] of a primarily intoxicated individual are consumed by conspecifics upon the return of the individual to aggregation in shelters and harborages. Commercial baits and active compounds can also vary in secondary killing characteristics. Rapidly acting compounds in baits may limit the return of pests to aggregations in harborages. Stejskal et al. [488] tested and classified baits into four categories in terms of their speed of action: (i) rapidly acting baits, less than 2 h (e.g., cypermethrin); (ii) quick-acting baits, 2–12 h (e.g., fipronil, chlorpyrifos, phenothrin (sumithrin or d-phenothrin)); and (iii) slow-acting baits, 12–72 h (hydramethylnon, boric acid).

Food baits are generally the preferred pesticide formulations, compared to insecticide sprays and dusts, due to their instrumentally undemanding application methods, potential for secondary transmission, low acute toxicity, minimal non-target effects, and low environmental contamination [477]. In contrast to pyrethroid sprays and physical disturbances, baits usually do not cause the budding and relocation of nests of ants such as *Monomorium pharaonis* (Linnaeus, 1758) [489]. However, a drawback of baits may be that an important level of resistance has developed in several baits of new generations (e.g., cockroaches [490], house flies [491,492]) within a few years of their commercial availability. One of the reasons is that bait efficacy may be associated with reduced pest feeding on baits [493,494]. Pesticide producers have to cope with a challenge that in the case of baits, unlike sprays or dusts, pests may develop (physiological/behavioral) resistance, not only towards active ingredients but also towards nontoxic food components, such as natural sugars [490]. Recent work by Wada-Katsumata and Schal [495] indicates that salivary digestion may protect (e.g., via oral hydrolysis of oligosaccharides, releasing glucose as a deterrent that causes food-bait rejection in sugar-resistant populations) the cockroach from ingesting toxic chemicals and thus could support the rapid evolution of behavioral and physiological resistance in cockroach populations.

### 4.2. Expandable Insecticide Foams (Baits and Contact Insecticides)

Foam-generating formulations of insecticide liquids (active ingredients, water-soluble polymer emulsifiers, etc.) are sold in pressurized containers (Figure 4L). After the rapid release of liquids from containers, these formulations mix with air and create foams that may be characterized as “dry” or “wet”, depending on the liquid–air ratio (https://www.pctonline.com/article/-formulations—fundamentals-of-foam/ (accessed on 24 June 2021)). Currently, commercially available foams mostly contain synthetic insecticides (against wasps and structural crawling insects) or repellents applied on human skin (against ticks and mosquitoes). Foams based on botanicals are available only as contact insecticides against urban or parasitic pests or as mosquito repellents. Foams are used as either (i) contact or (ii) bait-consumed insecticides (https://patents.google.com/ (accessed on 24 June 2021) patent/EP0289756A2).

(i) Insecticide foam baits are delivered via pressure cans. Aerosol-derived foam comprises edible foam carriers and food attractants. Portions of edible foam may be either eaten on-site or carried away into pest nests (https://patents.google.com/ (accessed on 24 June 2021) patent/EP0289756A2). (ii) As contact insecticides, expandable insecticide foams are applied to surfaces (Figure 4A,B) or internal spaces (Figure 4C), where foam expands rapidly. Most commercially available ready-to-use cans of foam have a 30:1 foam expansion ratio (https://www.mypmp.net/sponsoredcontent/the-scoop-on-foam/ (accessed on 24 June 2021)). The application methods and strategies include void filling, drill-and-treat application, crack-and-crevice treatment, application along narrow access passes, and application around technical piping and wiring (https://entomology.ca.uky.edu/ef614 (accessed on 24 June 2021); https://www.pctonline.com/article/-formulations--fundamentals-of-foam/ (accessed on 24 June 2021)). After effective application, the foam fills part or all of the target micro-space, and pests are either hit directly, via contact with a toxic foam filling, or during consequent passing. The main insecticidal effect of foam formulations is likely based on the “wrapping” of pests, thereby facilitating a high level of dermal contact. The enhanced foam effect may be associated with the cleaning behavior of cockroach pests, which consume a portion of the insecticidal foam during cleaning, similar to bait treatment (https://patents.google.com/patent/US4889710 (accessed on 24 June 2021)). Although foams can potentially affect hidden pests in the cavities of food processing rooms or warehouses, we did not find any published data on their usage or effectiveness on storage mites (Acari) or insects (Coleoptera, Lepidoptera, and Psocoptera).

### 4.3. Acaricide Gels and Coatings (Films, Nets) for Ham Protection

Mite infestation (e.g., *Tyrophagus putrescentiae* (Schrank)—Acari) of dry-cured hams (e.g., uncooked, cured, dried, and smoked or unsmoked pork products) during the product aging process is a problem, since it is hard or impossible to control these pests by means of traditional sprays, aerosols, or fumigants. As alternatives, there have been proposals to use natural acaricide compounds, modified atmospheres [496], and various physical coatings, such as vegetable oils or hot lard [497]. Recently, as alternatives, food-grade protective gel coatings (films) (Figure 4K) or nets (Figure 4J) with gels possessing acaricidal properties have also been suggested [498,499]. Such coatings must not affect the sensory properties of dry-cured hams but should allow water permeability [500]. A potential candidate compound is propylene glycol, which is completely miscible with water and many organic solvents and is used in cosmetic and pharmaceutical formulations. Zhao et al. [498] found that food coated with xanthan gum + 20% propylene glycol and carrageenan/propylene glycol alginate + 10% propylene glycol was effective in controlling mite infestations under laboratory conditions. Campbell et al. [499] demonstrated that ham nets treated with a food-grade coating of 1% propylene glycol alginate + 1% carrageenan + 40% propylene glycol tested in a commercial study provided significant protection not only against mites but also against undesired molds.

### 4.4. Gels, Gelatines, Starch Pastes, and Wax Polish Used for Residual Insecticide Pre-Treatment or Co-Treatment

The persistence and efficacy of insecticidal structural sprays depend on the quality and structure of the treated surfaces [332,401,426]. Porous surfaces may prevent the creation of sufficient insecticide deposits of droplets or to form an effective toxic film. Ceramic or painted surfaces do not substantially absorb liquids, hereas wood or bare brickwork have a high sorption capacity for many insecticide formulations. On such surfaces, insecticide dust or water-dispersed powders (WP) are preferred, since water is absorbed into the brickwork, leaving the insecticide on the surface [32]. As an alternative, co-treatment or pre-treatment of a surface by means of protective coatings has been proposed [501]. Hewlett [502] found that pre-treatment of cement with various gelatins greatly prolonged the toxic lifetime of films formed by several types of oil solution insecticides. Parkin and Hewlet [503] found that coating bricks with starch paste and water glass increased the activity of DDT and pyrethrins against *T. castaneum*. Tyler [504] incorporated carboxymethyl as a protective co-treatment in malathion sprays, which resulted in markedly improved persistence of the residual film on an alkaline cement substrate. Gudrups et al. [426] reported that permethrin applied to concrete with a wax polish coating provided the control of *R. dominica* for 14 days, whereas identically treated gloss-coated surfaces provided control for only 3 days. Hewlet [436] found that some petroleum oil films were highly toxic to tested weevils (*Sitophilus* sp.) when applied on cement pre-treated with gelatin. Although the latter finding may seem old and outdated, it may provide inspiration for enhancing the field efficacy of currently tested botanical oils and other types of natural insecticide compounds.

## 5. Insecticide Delivery in Solid Forms

### 5.1. Smoke-Generating Formulations (Chemically Activated or Ignition-Activated Pyrotechnic Smoke Generators, Cartridges, Tablets, and Canisters)

Active pesticide and biocide (i.e., disinsection and disinfection preparations [505]) ingredients can be effectively delivered to the target sites in the form of smoke. Insecticidal smoke is a form of dry aerosol [506] that is deliberately dispersed in the air as tiny solid micro-particles; the condensed smoke particles tend to aggregate as the concentration of smoke increases [324]. Apart from solid particles, the burning process also produces invisible gases (CO_2_, CO, SO_2_, etc.) that may have, depending on the concentration and length of exposure, some insecticidal action. Smokes are used, similarly to liquid aerosols, to directly target active or sedentary arthropod pests in enclosed environments. Cornwell [324] claimed that surfaces covered with finely divided smoke deposits can even cause the contact-mediated mortality of crawling insects such as cockroaches. Marke and Lilly [507] observed contact toxicity of DDT and gamma HCH on non-porous surfaces against *T. castaneum*.

**Figure 5:**
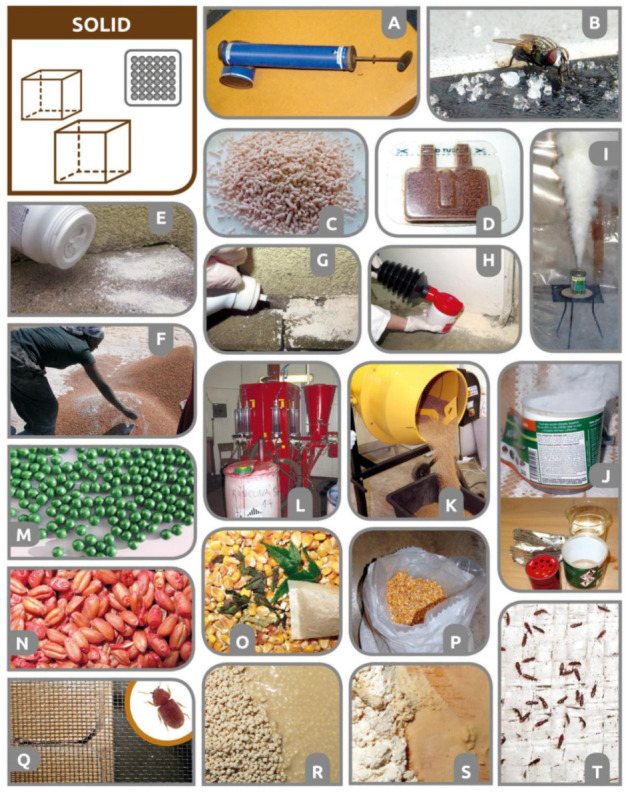
Solids. (**A**) Historical hand-pumped piston-gun: it was modified either for the application of aerosols (“Flit-gun”/“Fly-tox”) or as a sprayer or duster for the application of insecticide deposits; (**B**) application of baits in solid crystalloid form (sugar); (**C**) pelleted baits in bulk formulation; (**D**) granular baits enclosed in protective boxes; (**E**) duster container with a perforated lid applicator; (**F**) manual application of botanical ash/dust by low-income rural farmers; (**G**) duster container with an injection lid applicator; (**H**) visualization of barrier dusting; (**I**) ignition-activated smoke generator; (**J**) water-activated (chemically activated) smoke generator; (**K**) dust admixed with grain with the help of motorized or manually rotated drums; (**L**) seed treatment (dressing/coating and coloring with a warning dye); (**M**,**N**) various forms of dressed, coated, and colored seeds; (**O**) visualization of an admixture of grain kernels with solid traditional (fresh or dried parts of plants, e.g., neem) and new (tablets, granules, sachets) formulations of botanical insecticides; (**P**) visualization of a triple-layer hermetic bag with incorporated insecticide; (**Q**) visualization of a pest control net with incorporated insecticide; (**R**) solid granulated bait (**left**) and its slurry form (**right**); (**S**) solid inert dust (**left**) and its slurry form (**right**); (**T**) insecticide-/repellent-incorporated packages (photographs (**A**–**T**): V. Stejskal; R. Aulicky, T. Vendl).

There are two basic categories of smoke generators. The first category includes chemically activated smoke generators [506]; the example of a water-activated smoke formulation is visualized in Figure 5I. The second category includes pyrotechnic (ignition-activated) combustion smoke generators (Figure 5J) [38]. Deong et al. [506] claimed that many insecticidal compounds (DDT, chlordane, certain thiocyanates, methoxychlor, and benzene hexachloride) can be dispersed by chemically activated methods with negligible decomposition. In contrast, combustion and burning may cause partial decomposition of some active compounds [508]. The category of pyrotechnic formulations may be further separated into two subcategories: home-made dried organic natural materials and ready-to-use pyrotechnic combustion mixtures.

The first subcategory of pyrotechnic formulations comprises home-made dried organic natural materials containing natural botanical insecticides that are released during ignition. Busvine [91] considered such simple pyrotechnic formulations to be some of the oldest insecticide and disinfection agents. In ancient grain stores, the burning of plant materials in semi-hermetic underground storage pits provided combined toxic (releasing botanical insecticides + carbon monoxide) and hypoxic effects (releasing carbon dioxide) [152,509]. Tola et al. [122] compared various biomass materials (maize cob, maize stalk, cow dung, *Olea africana* wood or bark, *Maesa lanceolate* leaves, and charcoal—not fully burnt) for their potential to generate smoke with sufficient concentrations of CO and CO_2_ in stored grains. The results showed that smoke from dried maize stalk was superior in terms of the generation of high concentrations of CO (>2% vol) and CO_2_ (>11% vol), leaving less smell and flavor in the treated grain. More recently, it was found that pre-treatment with smoke (generated by burning cow dung cake) for 24 h increased the susceptibility of *R. dominica* to PH_3_ [510].

The second subcategory of pyrotechnic formulations consists of ready-to-use pyrotechnic combustion mixtures containing registered synthetic or natural compounds. Pyrotechnic combustion formulations contain insecticide mixed with a chemical flammable/combustible matrix (dusts, granules) that can be ignited to burn and produce a defined volume of dense white or gray smoke. Commercial mixtures are formulated as either quick-release or slow-release formulations. Coils and spirals are typical examples of slow-release smoke-releasing formulations [91]. Quick-release formulations—occasionally called “smoke bombs” [324] or fumigation canisters [511]—include tables, plastic containers, and metal cans (Figure 5I). Ignition of the smoke generator is ensured by a pre-installed wick or by insertion of a “non-sparkling sparkler”. The mixture must contain active ingredients that persist when exposed to high temperatures (e.g., pirimiphos-methyl, permethrin, cypermethrin, γ-HCH (i.e., gamma-hexachlorocyclohexane), or Lindane; sometimes incorrectly called benzene hexachloride or gamma BHC) [33]. Cornwell [324] considered smoke generators to be valuable formulations for use in poorly accessible areas such as cellars, technical ducts, and sewer channels. Munro [26] mentioned smoke generators as viable options for ship-hold treatment; he claimed that they provided reasonably uniform insecticide deposits over extensive surfaces. Freeman [32] and Herford [512] reported that smoke generators based on DDT or γ-HCH were commonly used in empty grain stores in the UK but warned that “*A smoke possesses poor powers of penetration and is quite useless for killing insects living inside foodstuffs; in fact, even a light covering of straw, sweepings or sacking is sufficient to afford effective protection to insects… insecticidal smokes deposit a useful toxic layer, especially on horizontal surfaces. The insecticidal value of this layer will depend largely on the likelihood of its becoming covered by dust and so rendered ineffective*“. In concordance with the latter claim, the FAO commodity storage manual [313] stated, “*Smoke generators can be used as a substitute for fogging in small and confined premises against moths and other flying insects. A limited effect may be obtained against crawling insects if higher dosage rates are used*.”

The most comprehensive study regarding the physical properties of insecticide smoke was conducted by Roff et al. [508]. These authors investigated burn characteristics, deposition patterns, pesticide air concentrations, and potential exposure to operators in three types of devices containing dicloran, permethrin, and red dye as active ingredients. Permethrin devices are designed to burn at lower temperatures (233 °C) than dicloran or red dye (283 °C and 392 °C, respectively). Pesticide air concentrations increased after firing, reaching a maximum determined by the room volume in approximately 10 min and decreasing exponentially as a result of ventilation and deposition. Approximately 50% of the pesticide active compound was consumed (degraded) during firing. The measured plume velocities above the fired pyrotechnic device were 0.5–1 m.s^−1^. The generated smokes had consistent particle sizes (0.5 and 3 microns) and contained approximately 50% pesticide and 50% ash.

Despite the widespread practical use of smoke generators in some empty commodity stores, silos, and food industry facilities, exact data based on controlled field validations are scarce [285,513]. Deong et al. [506] tested various experimental formulations of chemically generated dry smokes containing various insecticide compounds (DDT; thiocyanate, i.e., Lethane 384 or Lethane A-70; chlordane; toxaphene; lindane, i.e., γ-HCH) and their ratios (e.g., 20% DDT and 8% thiocyanate; 20% DDT and 5% chlordane). The authors [506] estimated the knockdown (of insects exposed in wire cages) and residual effects of various mixtures of insecticide smokes on *M. domestica*, *S. oryzae*, and *S. granarius* under laboratory and/or semi-field conditions. They also validated the efficacy of insecticide smoke (18% DDT, 5% chlordane, and 6% lindane; 24 h exposure) under field conditions in a railcar (cars were sealed for HCN fumigation) infested by *O. surinamensis* and *T. confusum* [506]. The authors found no immediate survival of pests following aerosol treatment and observed that smoke-treated grain (swept from the floor of railway cars) exhibited residual action on the tested insects for 6 months. Marke and Lilly [507] described the physical properties of the smoke deposits of DDT and gamma HCH and their toxicity to *T. castaneum*. On the treated surfaces, the γ-HCH smoke mixture was gradually deposited on the surface in the form of crystals and crystal aggregates, whereas DDT deposits had entirely different characteristics, since they initially did not form crystals but rather liquid drops. However, the consequent tarsal contact of *T. castaneum* with DDT liquid drops on glass also caused crystallization. On a non-absorbent surface, the DDT deposit appeared to retain its toxicity throughout the period of 30 days, but on an absorbent surface, its toxicity fell rapidly to a low level. The mortality of *T. castaneum* on HCH deposits was generally lower than that on the DDT-treated non-porous surface. More recently, Stejskal et al. [513] evaluated the biological efficacy of insecticide pyrethroid- and organophosphate-based smokes for German cockroach control and found that unobstructed smoke aerosol exposure may result in 100% cockroach mortality during 4 h of exposure. Stejskal et al. [48] also compared the activity of cypermethrin and pirimiphos-methyl applied as either a smoke or a ULV aerosol on four storage pests in one well-sealed experimental chamber and another in a large real-world store. It was found that both the airborne residues and the biological efficacy of smoke aerosols decreased over time more rapidly in the well-sealed chamber than in the unsealed large stores. Substantially lower residues of both insecticides were recorded for the smoke generator than for the ULV treatment in all of the exposed food commodities (flour, pea, oat flakes, rice, wheat, sunflower seed, and rape/canola seeds).

### 5.2. Baits Applied as Solid Dusts, Granules, or Semi-Solid Slurries

Practical users frequently refer to solid bait formulations as “stomach poisons” since they mainly enter the arthropod body via the digestive tract [91,324]. Solid baits for ant, fly, and cockroach control are delivered to the target locations in the form of dusts, dry granules, water-storing granules [514], blocks, tablets, crystals, etc. (Figure 5B,C). These baits are administered either freely or enclosed in ready-to-use containerized stations (i.e., plastic or metal boxes) (Figure 5D). In food industry facilities, baits in boxes enable regular checking, bait replenishment, and replacement or safe removal after usage. According to Tee and Lee [453], granular baits are designed for use outdoors against peri-domestic pest cockroaches; e.g., dry flowable powder baits are formulated for use in cracks and crevices to reach deep spaces, where the application of gel and paste is restricted. Solid granular baits for fly control can be alone or mixed with water to form slurry formulations (Figure 5R) [472], which increases bait attractiveness for some Diptera pests and enables their administration by spraying or brushing. The comparison of some currently used and physical formations (granular or liquid) of fly baits made by Parker et al. [472] revealed that “*significant degradation of baits was observed even when the baits were allowed to age in a covered outdoor environment. Baits were exposed to natural temperature and humidity levels and absorbed water readily. The hydrophilic nature of the baits may have contributed to either a degradation of the active ingredient, the attractive agent, or both. Quick degradation of baits, even in protected conditions, demonstrates the need for repeated reapplication of baits for maximum efficacy*.”

Historical solid cockroach and ant baits contained mainly inorganic (e.g., borax, boric acid, phosphorus, sodium fluoride) or organochlorine compounds, whereas the current solid commercial formulations are mainly based on active ingredients from neonicotinoid (imidacloprid) pyrrole (fipronil) groups [453]. Bait for wasp control may consist of a toxicant (fipronil, micro-encapsulated diazinon, avermectin, fenoxycarb, amidinohydrazone, etc.) incorporated into fresh or canned protein pieces based on fish, chicken, beef meat, or pet food meat mixtures [271,515,516,517]. Slow acting baits are based on insect growth regulators/disruptors (IGRs/IGDs) such as r/s-methoprene, s-methoprene, pyriproxyfen, etc. For example, for the control of *M. pharaonis* in buildings, granular solid baits were developed based on r/s-methoprene and s-methoprene [479,518,519]. For cockroach control, a solid granular bait containing novaluron (from the CSI (chitin synthesis inhibitor) group) was recently registered in the USA.

Baits containing new generations of active ingredients and attractive nontoxic bait components were shown to have high activity against many food industry pests, such as ants, cockroaches, and flies [520]. However, with the exception of a bait (based on cassava + insecticide) used for the management of the coffee bean weevil (*Araecerus fasciculatus* (De Geer)—Antribidae) in stored cocoa [521], we were not able to find any other published reports on solid (or semi-solid) bait usage to control stored-product insects or mites. Solid anticoagulant baits are the main rodenticide formulations for rodent control in stores and food industry facilities. Since anticoagulants do not show insecticidal activity, unattended rodent baits frequently serve as a medium for the development of stored-product beetles [522] and as occasional food for arthropod scavengers such as cockroaches [523]. Because of this risk, there have been attempts to combine rodenticide and insecticide-cockroach baits [524]. However, currently, this approach is extremely demanding legislatively and economically costly due to the necessity of the registration of two active ingredients in a single bait product.

### 5.3. Application of Synthetic or Natural Organic and Inorganic Insecticide Dusts (Structural, Grain Admixture)

Insecticide dusts usually consist of uniform spectra of small (5 μm) solid particles, since fine dusts are more readily picked up by arthropods than coarse dusts [324]. Irrespective of the dust size spectra, active ingredient, or mode of action (i.e., neurolytic or desiccation), the activity of all types of insecticide dust requires dry conditions [525,526]. The delivery of insecticides and acaricides in the form of dust is among the most ancient methods used to control insect pests [527,528,529]. The industrial use of inert dust for stored-product protection was first suggested by Friedrich Zacher [530,531] in Germany more than 100 years ago. Consequently, scientific and practical approaches for the application of insecticide dusts were further elaborated and extended in the UK [532,533], USA [534,535,536], and in several other countries and regions during the first decades of the previous century. Two commercial products, known as “Naaki” (in Germany) and “Neosyl” (in England), were marketed for stored-product protection in the 1930s and early 40s [529,532]. In the historical products, quartz was the active ingredient, which is now classified as a human carcinogen. Therefore, the current commercial DE formulations are mainly made up of amorphous silica and contain little (up to 4%) or zero crystalline silica [529].

Munro [26] simply grouped insecticidal dusts into only two categories: “chemical dusts” and “inert dusts”. Cornwell [324] called chemical dusts “insorbicides” (i.e., “formulations that are not sorbed by the treated surface”), which are prepared by precipitating insecticide onto kaolin talc and starch. Early inorganic chemical dust used for surface/structural treatment included a limited number of compounds (lead arsenate, cryolite, boric acid, borax, sodium fluoride, or sodium fluorosilicate, etc. [324,363]) with long residual activity. Boric acid dust is still considered a useful component of some IPM programs for cockroach control [537], among other reasons because they have not yet developed resistance to this substance. These dusts penetrated arthropod bodies via unsclerotized parts of their integument but also acted as stomach poisons after their ingestion during insect grooming and autocleaning [324]. According to Shepard [211], copper carbonate dust was applied (two ounces per bushel) to stored wheat seeds not intended for animal or human consumption in the past. Recently, the idea of using metal-based compounds has returned in the innovative form of nanomaterials. For example, copper [538] or silver [539] nanoparticles were suggested as insecticides for the control of the stored-product beetle *T. castaneum*. Rahel et al. [540] tested the acaricidal effect of chitosan and chitosan/metal adducts with Ag(+), Zn(2+), and Cu(2+) on the mites *Acarus siro* (Linnaeus), *Dermatophagoides farina* Hughes*, Dermatophagoides pteronyssinus* (Trouessart) and *Tyrophagus putrescentiae* (Schrank) (Acari), with promising results.

Currently, commercial dust formulations are usually classified into three basic categories: (i) inorganic or organic chemical (i.e., conventional) insecticide dusts, (ii) inert dusts of natural or synthetic origin [529,541,542,543,544], or (iii) various combinations of natural and synthetic dusts (e.g., kaolin/diatomite + DDT/HCH-lindane [545]). Organic insecticide dusts historically have contained a broad variety of neurotoxically active compounds (e.g., organochlorines, carbamates, organophosphates, pyrethroids, and natural pyrethrum extracts, piperonyl butoxide). Most organic insecticide dusts are ready-to-use formulations with 1–5% concentrations of the specific active ingredient [324]. Inert dust includes multiple compounds with abrasive, desiccating, and suffocating modes of action [536,546]. These different modes of action explain why organic insecticide dusts generally require the application of lower deposition amounts than inert dusts. According to Golob and Webley [528], inert dusts are broadly divided into synthetic materials (silica aerogels) and a variety of materials of natural origin. Natural products include non-silica dusts (e.g., katel-sous, i.e., rock phosphate and ground sulfur; lime; limestone; and common salt), diatomaceous earths (i.e., diatomite, composed mainly of amorphous hydrated silica, aluminum, iron oxide, magnesium, sodium, and lime), and other silica-containing materials (sand, kaolin, clays, and zeolites). It is important to distinguish these groups since they affect the application dosage—the lowest dose is required for silica aerogels, followed by diatomaceous earths (0.1 g *w/w*), and the largest doses and quantities (5% by weight) are required for sand, kaolin, ash, and clays [528]. Since diatomaceous earth (DE) [547,548] is a natural product, its physical and biological activity after application may differ according to its geographical origin [525,526]. In some commercial or experimental preparations, inert dust is mixed with biorational products (IGDs/IGRs [549], spinosines [40,550]) or neurotoxic insecticide compounds (e.g., deltamethrin [551]). The effect of inert dust can be increased by combination with biological or physical stressors [552], such as structural heat treatment. However, it should be emphasized that the idea of combining various natural dust or botanical powder compounds and their formulations to achieve higher efficacy in controlling storage and urban pests is not new [361]. For example, Dove [437] noted that ground dust impregnated with pyrethrum extracts is more effective than ground pyrethrum flowers.

#### 5.3.1. Dusts Applied as Surface and Structural Treatments

**Figure 6:**
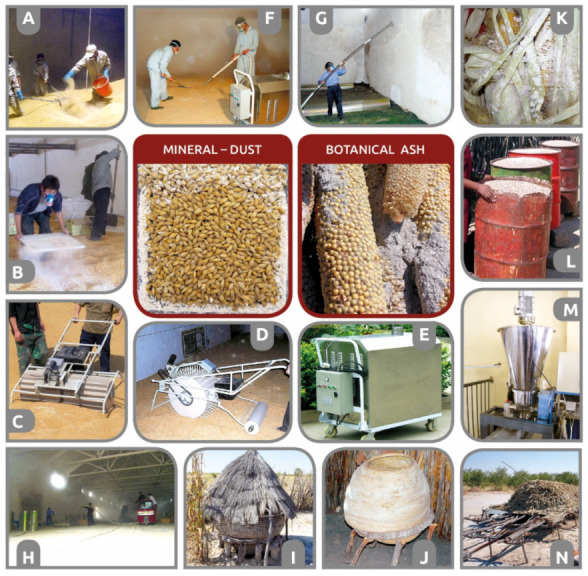
(**A**,**B**) Various types of manual applications of inert dusts for surface and subsurface grain treatment in horizontal stores; (**C**,**D**) two types of motorized blenders for surface and subsurface grain treatment in horizontal stores (ASAG-Beijing); (**E**) one purpose-built automatic dust applicator machine (ASAG-Beijing); (**F**) surface and subsurface manual application with the help of an automatic dusting machine; (**G**) application of dust on the wall of a horizontal store with the help of a dusting machine; (**H**) broadcast structural treatment of an empty store with inert dust; (**I**,**J**) surface of empty storage wickerwork baskets treated using inert dust or ash; (**K**) visualization of the layer treatment of maize cobs using diatomaceous earth dust; (**L**) grain stored in drums treated using botanical ash; (**M**) dust applicator for continual grain treatment on a conveyor belt inside a silo building (ASAG-Beijing); (**N**) short-term outdoor storage of commodities treated using botanical ash. Note: Photographs (**A**–**H**) were kindly provided by Prof. Dr. Cao Yang and Prof. Dr. Yanyu Li (Academy of National Food and Strategic Reserves Administration, ANFSRA and ASAG Beijing) solely for the purpose of this review (photographs (**I**–**N**): V. Stejskal; T. Vendl).

Insecticide dust treatment is one of the most common methods used for the application of insecticides in empty commodity stores or in the food industry, especially where spray formulations cannot be applied. Dusts are not absorbed into porous surfaces, but some of them may be more repellent than many spray formulations [324]. Typically, dust is used for the structural treatment of empty storage materials, traditional storage wicker baskets, transportation freight containers [57], and facilities via surface and crack-and-crevice treatments [529,553,554]. Diatomaceous earth and ash are suited for the internal and external treatment of the complex wickerwork surfaces of empty traditional baskets used for grain or legume storage in developing countries (Figure 6I,J) [555]. The aim is to create highly effective insecticidal deposits that either prevent (repel) the entry of pests or kill the pests after they climb on the surfaces. Methods of the structural application of dusts are similar to those already described for spray insecticides in this review: surface application, barrier treatment, spot treatment, and crack-and-crevice treatment injection [553,554]. However, the methods and equipment used for their application differ. There are small simple application devices (e.g., plastic containers with perforated lids (Figure 5E) or plastic injectors (Figure 5G), bulb dusters, and piston-gun dusters, (Figure 5A)), as well as more complex manual (Figure 5H) or electrical devices for barrier applications and/or injection into internal structural voids. Powerful industrial application equipment (Figure 6G,H) is used for the broadcast application of dust to the floors and walls of cereal and legume warehouses. Cao et al. [556] described the construction and use of purpose-built dusters (sprayers) for the control of stored grain insect pests in a large empty warehouse (Figure 6E,F). When the dustiness of inert dust could be a problem during its application, a slurry formulation may be selected as a solution to this issue (Figure 5S) [553]. Slurries may be distributed by means of special industrial sprayers for broadcast application or via ready-to-use total release containers with compressed propellants for crack-and-crevice treatment or applications in different types of surfaces. Equipment producing electrostatically charged dust particles may also enable more targeted and efficient dust applications [557,558].

#### 5.3.2. Dusts Applied as Commodity Admixture Protectants

The general advantage of dust-type protectants is that they can be simply and evenly mixed with various stored commodities, even in situations where there is a lack of technology for the continuous treatment of grain moving on conveyor belts (Figure 6B) [37]. This is likely one of the main reasons why synthetic and natural insecticide dusts have been one of the most widespread and used forms of admixture grain protectants. The active ingredients of conventional synthetic insecticides have historically been selected mainly from chemical groups such as organochlorines (lindane [559]), organophosphates (malathion [560], pirimiphos-methyl), pyrethroids (deltamethrin [551]), and and pyrethrins combined with piperonyl butoxide [561]. The problems associated with unwanted insecticide residues, with decreasing maximum residue limits (MRLs) for exported/imported commodities, and with the development of resistance (namely, malathion and phosphine, as well as other active ingredients) facilitated the development and usage of inert dust formulations (Figure 6), which might partially replace conventional chemicals. For commodity treatment, a combination of food-grade inert dust with natural compounds as synergists has also been tested [562]. Campolo et al. [563] reported that kaolin admixed with *Citrus sinensis* peel essential oil might be a viable alternative to the chemical pesticides commonly used in wheat pest management. This combination caused, in a synergetic way, not only significant mortality of *R. dominica* but also reduced growth of yeasts, molds, and total mesophilic aerobic bacteria. Most recently, Korunic et al. [564] proposed cocktails of inert dust with pyrethrum, amorphous silica gel, flax oil, lavandin essential oil, and inactivated yeast. However, inert dust, such as diatomaceous earth, may affect the grain bulk density and surface kernel friction [565]. A high dose of dust evenly admixed over the entire profile of grain commodities may thus result in problems with the discharge of these commodities from silos and further manipulation for transportation. It was therefore suggested by several authors [556] that a limited extent/volume of grain can be treated, i.e., surface/subsurface (top-dressing), or multilayer treatment only (Figure 6A–D,F). For example, partial commodity (e.g., maize cobs; Figure 6K) treatment can be achieved via gradual (“sandwich-type”) treatment of consequently loaded layers of grain or maize cobs into stores, drums, or wicker baskets [313]. Another approach is to mix smaller grain piles with dust part-by-part with a shovel or apply dust into portions of the commodity using motorized or manually rotated barrels (Figure 5K). Dusts can be manually applied by hand with gloves (Figure 6A) or by trays/boards/sieves (Figure 6B) to the surface of the grain (“top-dressing”) and then mixed under the grain surface with a shovel or rake (Figure 6F). A new generation of motorized surface applicators and blenders of dusts has been developed and introduced into practice in China (Figure 6C,D,F). Electrostatic powder was reported to be used to reduce the amount of pirimiphos-methyl applied to grain for effective pest control [558].

Partial commodity treatment, top-dressing treatment in particular, is inherently associated with a varying risk of incomplete efficacy. As noted by Arthur [566], both dust and spray top-dressing may offer insects ample opportunities to penetrate through the treated surface into the untreated portion of the grain. Vardeman et al. [567], in a study with diatomaceous earth (DE), reported that stored-product insects may penetrate through a dust-treated surface in a grain mass in a physiological state that enables them to oviposit in the untreated layers before they die.

### 5.4. Botanical Ash, Dust, Powders, Particles, Leaves, Phyto-Tablets, and Sachets

Currently, there is great worldwide research interest in the use of innovative traditional methods of protection against storage pests, especially the use of botanical substances. The most studied substances are plant oil extracts [568,569]. However, in rural areas, botanical ash (Figure 5L,N) [29,555,570] or whole parts of dried or fresh plants (Figure 5O) have also been mixed with grain as protectants or repellents [528,571]. Goudoungou et al. [572] obtained promising insecticide results with a binary combination of leaf powder (*Plectranthus glandulosus)* and wood ash *(Hymenocardia acida*). Haq et al. [571] tested the repellent efficacy of leaf admixtures (5% wheat grain) of *Eucalyptus* sp*., Bougainvillea glabra, Azadirachta indica, Saraca indica*, and *Ricinus communis* to prevent the entry of the red flour beetle *T. castaneum*. Another ancient protective approach involves a stored kernel admixture containing dust/powder and oil mixtures from different dried plants [573] or containing a mixture of botanicals and cow dung ash as a carrier [574]. However, contamination of the commodity with difficult-to-remove organic dust, sometimes associated with a persisting aroma, can be a problem. New research and new application formulations have surmounted these obstacles. The first formulations that have been designed to be easily removed after the admixture treatment of a commodity are botanical dust tablets, granules, and phyto-tablets [575,576]. Other formulations that physically separate the bio-insecticide residues from the treated commodity include sachets (bags) filled with botanicals. These sachets are fabricated from a porous paper material, allowing the diffusion of substances from the sachet into the commodities. Chang et al. [577] showed that sachets containing 2% allyl mercaptan showed repellent effects on *S. oryzae* during 48 h of exposure and no undesirable changes in the sensory properties of rice both before and after cooking.

### 5.5. Incorporated Insecticides: Seed Dressings, Toxic and Edible Coatings and Films

Seeds with industrially incorporated pesticides are usually treated by various warning dyes (Figure 5L–N). They are supplied as ready-to-use pesticide-coated seeds to end users by seed production companies [36,285]. From the user’s point of view, these seeds may therefore be considered a “solid” form of insecticide because, in principle, they act as a special form of ready-to-use bait. The incorporated pesticide dressings and coatings are used most commonly as both fungicides and insecticides for seed or seedling protection (Figure 5M). For example, a bendiocarb seed dressing served both as a guard against attacks by frit fly and wireworm larvae and as a bird repellent (https://www.sciencedirect.com/topics/agricultural-and-biological-sciences/seed-dressings (accessed on 24 June 2021)). Although such seeds contain a broad spectrum of pesticides, little information is available on the effects of various seed coatings and dressings on stored-product pests. The rare exception is a publication by Zdarkova et al. [578] containing information regarding a fungicidal seed dressing used in the Czech Republic in the 1960s—based on the substance methylmercuric dicyanamide—which was also effective in suppressing storage mites. Although uncoated beet seeds are prone to infestation by storage insects and mites, it appears that the recent widespread adoption and use of coated sugar beet seeds in farming practice has solved this problem [579]. Due to their toxicity, most dressings are not allowed for the treatment of kernels for human or animal consumption. Recently [580], a quality protective (oxidative stability) edible film coating containing rosemary extract for use on sunflower kernels was designed. It remains to be determined whether this or a similar type of protective edible coating also impacts the development of stored-product arthropods infesting stored oil seeds.

### 5.6. Insecticide Incorporated/Impregnated Bags, Packaging Foils and Packages (“Active Packaging”)

**Impregnated food packaging or wrapping foils**. Many storage pests have the ability to penetrate the packaging or enter openings in the packaging of finished foods and various bags containing agricultural commodities. After the distribution of food to retail chains and to final consumers, its protection is completely out of the reach of food producers, although a multi-month guarantee of unaltered quality must be provided. Therefore, manufacturers have long sought to produce affordable packaging that is resistant to the penetration of harmful arthropods [288]. In addition to physically resistant multilayer packaging barriers, several forms of chemical protection have been investigated. One such method is the surface application of residual insecticidal sprays [292] or dusts [581]. An alternative and more promising method of food product/commodity protection is the incorporation of a protective substance into the packaging structure. Substances effectively incorporated into packaging (Figure 5T) then repel, hormonally disrupt [582], or directly kill pest invaders. Both synthetic and botanical insecticides have been tested as substances for incorporation into packaging [583]. Recently Marsin et al. [584] summarized the compounds and plants documented as plant repellents used in food packaging: pyrethrum (*Chrysanthemum* sp.), neem (*Azadirachta indica*), thyme (*Thymus vulgaris*), cinnamon (*Cinnamomum* sp.), citronella (*Cymbopogon nardus*), garlic (*Allium sativum*), pine (*Pinus sylvestris*), oregano (*Origanum vulgare*), rosemary (*Salvia rosmarinus*), ginger (*Zingiber officinale*), black pepper (*Piper nigrum*), onion (*Allium cepa*), fennel (*Foeniculum vulgare*), lavender (*Lavandula angustifolia*), peppermint (*Mentha piperita*), geranium (*Geranium maculatum*), palmarosa (*Cymbopogon martini*), eucalyptus (*Eucalyptus globulus*), and bergamot (*Citrus bergamia*).

**Methods of impregnation of foils for food packaging by EOs**. Impregnation can be based on non-encapsulated, microencapsulated, or nanoencapsulated EOs for a controlled release of the active ingredient. Two basic impregnation techniques include (i) surface or subsurface foil **coating** or (ii) **incorporation** into a foil matrix. Marsin et al. [584] stated that the surface coating procedure may involve dip, drop, spray, print, laminate, and electrospin procedures, whereas incorporation of a repellent may be accomplished by casting, blow film extrusion, or compression. Arthur [397] suggested a standard methodology for evaluating insect growth regulators/disruptors (IGRs/IGDs) on packaging films. At present, it is not possible to incorporate insecticides into food/commodity packaging without registration for this purpose. The reason is the risk of migration of insecticides from packaging into food or commodities. In the last decade, the number of scientific works on “active packaging” development has increased [585], but only a few commercial preparations are available, and their application in practice is rather scarce [585,586]. We did not find any published information on industrially used wrapping foils (“tertiary packages”) with the incorporated (registered) repellents or insecticide protectants.

**Impregnated commodity storage bags.** Insecticide incorporation methods are not only used for packaging protection of finished food products but have also been recently developed for use in smaller packaging (bags) (Figure 5P) intended for the safe storage of commodities on farms in rural areas [583]. To increase the level of protection, insecticide-incorporated coatings are considered to be combined with multilayer hermetic bags. Some of the suggested storage polypropylene bags may contain pyrethroids incorporated into the bag fabric (e.g., ZeroFly—3 mg deltamethrin/kg [192]).

### 5.7. Insecticides Incorporated in Nets and Nettings

To prevent the infestation of commodities and food without the need to directly treat sensitive commodities (i.e., the risk of pesticide residues) or primary packaging (i.e., the risk of insecticide migration from packaging), the concept of protective barrier nets with incorporated insecticides was established. Nets exhibit either repellent [587] or lethal [588] effects on pests. Historically, insecticide-impregnated nets originated with long-used malaria prophylactic products for the protection of humans against mosquito bites. Recently, mesh insecticidal products have been produced to protect expensive food products against storage pests (Figure 5Q). Specifically, Agrafioti et al. [589] tested the efficacy of nets coated with SiO_2_ dust nanoparticles under different pre- and post-harvest application scenarios. Polyester insecticide-impregnated net technologies, such as alpha-cypermethrin-coated polyester nets (Carifend^®^), were suggested to protect tobacco products from the cigarette beetle *Lasioderma serricorne* (F.) (Ptinidae) and the tobacco moth *E. elutella*. Rumbos et al. [588] found under laboratory conditions that this product could provide a satisfactory level of protection for stored tobacco against both species, even after brief exposure to the pyrethroid-coated net. Research on the activity of these nets on other pest species [590] and their behavior is currently underway [587,591], with very promising results. To extend this line of research, Andriessen et al. [592] proposed an innovative type of netting treated with an electrostatic coating that binds insecticidal particles through polarity; although this electrostatic netting led to decreased amounts of insecticide, it showed enhanced bioavailability upon contact with the insect. The authors indicated that practical applications included the use of electrostatic coatings on walls or eave curtains and in trapping/contamination devices.

## 6. Conclusions

**Two directions: the development of new technologies and the optimization of classical technologies.** The aim of this review was to provide an overview of traditional, new, and emerging methods for the application of gas, liquid, gel, and solid physical insecticide formulations to control stored-product and food industry urban pests. There are two main ways to characterize the current state and development of new protective measures for stored-product protection. The first aspect highlights the modification and optimization of traditional chemical approaches and formulations. The second aspect emphasizes that modern stored-product protection should be “greener” and based on natural resources, e.g., the utilization of atmospheric inert gases (N_2_ or CO_2_), inert dust, and botanical preparations (e.g., pyrethrum, neem oil). Additionally, some safe industrial wastes, such as filter cakes and triplex powders (by-products of aluminum sulfate and soap factories in Ethiopia), have been tested as insecticides [593]. Recently, hydrogel bait formulations [455,456], artificial insecticide sweeteners [276], and dRNA-based disruptors [283] have been suggested as new or emerging smart technologies for the establishment of new generations of food baits.

At the worldwide scale, as grain protectants, some more environmentally friendly options include industrially produced reduced-risk (low-risk) compounds, such as insect growth regulators and disruptors (IGRs/IGDs) or compounds derived from fungi or bacteria (e.g., spinosad—based on a fermented product from the soil actinomycete *Saccharopolyspora spinose* Mertz and Yao [219]), or inert dusts. Since IGRs have not been widely available for grain protection in Europe, a new project (EU—HORIZON 2020—novIGRain) has been established with the aim of developing the first European IGR-based grain larvicide protectant applied as a ULV spray [252]. This may provide a viable alternative or complement to some neurotoxic-insecticide grain protectants or to some grain fumigants (e.g., PH_3_) to which resistance may be on the increase [10,54,594].

It has been proposed that naturally-based pest management approaches (methods, agents, compounds, etc.) should be aggregated under the general term “bioprotection” [595]. Bioprotection should be discriminated from biorational pest management since the latter may include low-toxicity (low-risk) or inert synthetic/unnatural compounds [237]. According to Stenberg [595], the so-called bioprotection umbrella encompasses two broad groups that include either non-living nature-based substances or living biocontrol agents. Apart from avoiding toxic residues, many bioprotection and biorational methods do not generally alter the chemical properties of the treated commodities. According to Phillips and Throne [237], biorational approaches are among the most promising methods for the protection of stored commodities since not only are they environmentally safer but—as a recent research project revealed [192]—some of the bioprotection-based technologies might even outperform traditional chemical treatments in terms of their on-farm efficacy. For example, in a large-scale study from Zimbabwe, Mubayiwa et al. [152] demonstrated that the protection of smallholder-stored commodities using hermetic treatments (i.e., hermetic multilayer bags) resulted in lower commodity weight losses (<3%) than pesticide-based treatment protection (3.7–14.2%). The authors of a pilot study [192] concurrently found that *T. castaneum* developed in a pesticide-treated commodity containing deltamethrin and fenitrothion, whereas *S. cerealella* developed in a pesticide-treated commodity containing pirimiphos-methyl and thiamethoxam. In recent decades, there has been exponentially increasing interest in the research of botanical insecticides (insecticidal effects, repellency, antifeedant action, etc.) in the field (i.e., agricultural), urban and stored pest control [596]. Natural botanical compounds and formulations have been considered as non-synthetic chemical control solutions for organic food production in developed countries and as affordable home-made insecticides in developing countries [225]. However, with some exceptions (e.g., pyrethrum), botanical extracts are still awaiting for wider industrial acceptance. Among others, the following factors are currently acting as barriers for their wider adoption: registration costs, insufficient field efficacy data, and a lack of application formulations ensuring stability of the active ingredient and effective delivery to the target sites [69,70,225]. Potential producers of natural products are also facing a problem regarding how to ensure a defined content and concentration of active ingredients in plant materials, as well as to reduce the batch-to-batch variability in the preparation process [225]. Murdock et al. [29] emphasized that the adoption of natural insecticides might not just be a matter of technical feasibility, efficacy, and cost-effectiveness, but should also include local cultural perspectives. The authors demonstrated this aspect with the example of botanical ash used as a commodity protectant. The attitude towards this low-cost, readily available, safe, and environmentally friendly preparation was not found to be positive among smallholder farmers in all cases, since some rural communities associated ash with a symbol of death [29].

Another trend is the renewed research interest in and optimization of classical chemical methods, such as insecticide baits, foams, aerosols, sprays, and fumigants, as well as the feasibility of integrating these methods with inert gases, natural insecticides, and combined biorational application scenarios [237]. In this context, there are published arrays of promising results concerning binary or multiple combinations of various groups of active ingredients used as fumigants, aerosols, sprays, dusts, and baits (push-and-pull) [40,387,552]. These combinations may be viewed as opportunities to overcome the weaknesses and exploit the strengths of individual insecticides [127]. Daglish et al. [40] noticed that many novel approaches (new active ingredients and combinatory uses) are still in the stage of research without reaching the registration stage. In addition to binary sprays and dusts, insecticide-impregnated, -treated, or -coated hermetic bags have shown satisfactory performance as safe methods for commodity storage in developing countries [30,192]. One type of triple bag was shown to be usable not only for safe storage but also for effective phosphine fumigation [197]. There is also considerable potential for these methods in the packaging of various types of durable food [286]. The combination of impregnated and hermetic plastics is expected to provide promising results, especially for long-term storage in developing countries, and should therefore be examined in more detail. Moreover, there are different techniques available that can be used to enhance the attractiveness of baits or similar formulations, an approach known as the “pheromone-assisted baiting technique”, typically similar to the “lure and kill” or “attracticide” approach; for a review of this technique see, e.g., [597,598]. In addition to these methods, biodegradable acaricide gel coatings and nets may provide a viable alternative to fumigation and concomitant residues in commodities of animal origin, such as dried ham products [599]. Micro- or nanoparticles merit additional investigation, as they already play an important role in crop protection, whereas some compounds have been commercialized for use in the post-harvest stages of agricultural commodities. Two characteristic paradigms of these materials are diatomaceous earths and zeolites [541,600]. Furthermore, some of these materials can be used with success as binary combinations with botanicals (essential oils), insecticides, or entomopathogens [601,602,603,604].

**Historical inspiration and lessons:** In the 1960s, Scott and Lettig [363] expressed an opinion that the gradual development of insecticide formulations and compounds could be viewed as a constant historical search for an “ideal insecticide” or a so-called “magic bullet” or “magic potion”. The desired properties of such an ideal insecticide formulation include, among others, low cost, simple application, high pest control efficacy, and low toxicity to humans and non-target organisms. Scott and Lettig [363] described the historical search for such an “insecticide magic potion” on the example of house fly control, based on four consequently appearing basic pillars or ideas: *“(i) The first idea—poisonous minerals. (ii) The second idea—poisonous plants. (iii) The third idea—poisonous gases. (iv) The fourth idea—synthetic organic insecticides.”* The advent of synthetic insecticides (particularly organochlorines) led to inexpensive, highly effective, and stable substances that were not very toxic to humans. This success raised the general hope that the goal of achieving an insecticide “magic bullet” had been reached. Nevertheless, soon after the wider adoption of synthetic insecticides, a great disillusionment occurred when multiple pest species developed resistance to a wide range of chemical compounds. It was thus learned in the hard way that synthetic insecticides, used alone without the support of the other IPM components, were not the desired “magic bullets” capable of sustainably solving all pest problems. However, the process of transition from ancient to modern insecticide formulations and advanced methods of application led to the accumulation of a vast amount of valuable experiences and technological knowledge that should not be forgotten. Our review demonstrates, from historical data, that the current problems concerning the use of natural insecticides (variable composition and quality due to home-made preparation, photo-instability, short residual action, aromatic issues, etc.) echo some of the problems that emerged nearly 100 years ago in classical chemical storage and urban pest control [26,52,359]. Current researchers considering the formulation of natural products may learn from historical experience relating to the formulation and application of inorganic or organic synthetic insecticides. For example, in the 1940s, Murphy [364] and McDaniel [361] were among the first to propose or recommend a combination of natural and/or synthetic organic insecticides to increase their efficacy or compensate for the short residual action of botanical extracts. Early synthetic fumigants were saturated in porous discs and enclosed in porous satchels before their insertion into the treated commodity [52]. Similarly, while the use of some natural botanical agents may not be as feasible as direct applications on the commodity, they have been formulated as tablets [576] and satchels [577]. Sorption generally decreases the activity of gases and volatile compounds, which may be an important issue in terms of the decreased activity of some botanical compounds. However, following the sorption step, the gradual intensive desorption of an active compound may even prolong insecticide treatment, as demonstrated in the case of some volatile organophosphate insecticides [213]. Since commodity sorption may be a serious limiting factor even for classical synthetic fumigants (e.g., complete sorption may occur in ethyl formate in 24 h), split or forced fumigant application with CO_2_ has been suggested to partly minimize these problems [127]. The shorter sorption times and longer stability of early residual sprays was achieved through a special co-treatment or pre-treatment of the treated surface with oil or another protective coating [501]. The authors of this review believe that the few selected examples mentioned above indicate that historical experiences and application approaches have certain potential that deserves to be explored as a new inspiration for the application technology of modern generations of naturally based pesticide formulations.

**Qualification, education, and the adoption of new formulations and technologies**. A major downside of the majority of the novel techniques presented in this review may be that they are more expensive and more demanding in terms of personnel qualifications or training in terms of their application than the conventionally applied contact insecticides or fumigants. The increased cost for the utilization of some of these formulations is justified by the type of commodity; i.e., high-value commodities, such as dried fruit, herbs, and tobacco are good candidates for the use of expensive methods, such as controlled or modified atmospheres. However, other commodities, such as raw grains, may not be the target commodities for these methods. Hence, the economic feasibility of each method should be considered based on its cost, which, in practice, is of equal importance to its effectiveness and environmental compatibility profile. Unless a careful economic analysis is undertaken for each case scenario, i.e., the commodity, target species and type of facility, any data provided on the efficacy of certain methods may be unrealistic.

## Figures and Tables

**Table 1:** Categories and subcategories of insecticides that are currently in use in stored-product protection.

Category of Insecticide Formulation and Methods for Delivery to Target	Subcategory (Type) of the Insecticide Application Formulation
Vapors and gases	Vaporization and sublimation (cold, thermal, “residual fumigation“, etc.)
	Toxic gases (released from solid, liquid, or gas formulations)
	Inert gases (hypoxic/anoxic atmospheres)
Liquids	Admixture, dressing, dipping, and impregnation treatments of grain
	Coatings, paintings, and lacquers (structural surface treatments)
	Liquid baits
	Liquid droplets in air delivered as space treatment by aerosols, thermal or cold/ULV/fogs or mists
	Insecticide deposits on surface delivered as spray (indirect residual treatment)
	Direct treatment of arthropods by sprays
Foams, gels, and pastes	Expandable foams as insecticide barriers and cavity fillers
	Foam baits
	Gel, hydrogel, and paste baits
	Protective food gel coatings and layers on meat and cheese
Solids	Smokes (solid aerosols)
	Solid baits
	Dust, slurries, powders, ash, and nanoparticles
	Insecticides incorporated into protective nets
	Insecticide impregnation of packages (incorporation into the matrix/surface coating)
